# tRNA functional signatures classify plastids as late-branching cyanobacteria

**DOI:** 10.1186/s12862-019-1552-7

**Published:** 2019-12-09

**Authors:** Travis J Lawrence, Katherine CH Amrine, Wesley D Swingley, David H Ardell

**Affiliations:** 1 Biosciences Division, Oak Ridge National Laboratory, P.O. Box 2008, Oak Ridge, TN, 37831 USA; 2 Quantitative and Systems Biology Program, University of California, Merced, 5200 North Lake Rd., Merced, CA, 95343 USA; 3 Insight Data Science, 500 3rd St., San Francisco, CA, 94107 USA; 4 Department of Biological Sciences, Northern Illinois University, 1425 Lincoln Hwy., DeKalb, IL, 60115 USA; 5 Molecular and Cell Biology, School of Natural Sciences, University of California, Merced, 5200 North Lake Rd., Merced, CA, 95343 USA

**Keywords:** Plastids, tRNAs, Cyanobacteria, Primary endosymbiosis, Machine learning

## Abstract

**Background:**

Eukaryotes acquired the trait of oxygenic photosynthesis through endosymbiosis of the cyanobacterial progenitor of plastid organelles. Despite recent advances in the phylogenomics of Cyanobacteria, the phylogenetic root of plastids remains controversial. Although a single origin of plastids by endosymbiosis is broadly supported, recent phylogenomic studies are contradictory on whether plastids branch early or late within Cyanobacteria. One underlying cause may be poor fit of evolutionary models to complex phylogenomic data.

**Results:**

Using Posterior Predictive Analysis, we show that recently applied evolutionary models poorly fit three phylogenomic datasets curated from cyanobacteria and plastid genomes because of heterogeneities in both substitution processes across sites and of compositions across lineages. To circumvent these sources of bias, we developed CYANO-MLP, a machine learning algorithm that consistently and accurately phylogenetically classifies (“phyloclassifies”) cyanobacterial genomes to their clade of origin based on bioinformatically predicted function-informative features in tRNA gene complements. Classification of cyanobacterial genomes with CYANO-MLP is accurate and robust to deletion of clades, unbalanced sampling, and compositional heterogeneity in input tRNA data. CYANO-MLP consistently classifies plastid genomes into a late-branching cyanobacterial sub-clade containing single-cell, starch-producing, nitrogen-fixing ecotypes, consistent with metabolic and gene transfer data.

**Conclusions:**

Phylogenomic data of cyanobacteria and plastids exhibit both site-process heterogeneities and compositional heterogeneities across lineages. These aspects of the data require careful modeling to avoid bias in phylogenomic estimation. Furthermore, we show that amino acid recoding strategies may be insufficient to mitigate bias from compositional heterogeneities. However, the combination of our novel tRNA-specific strategy with machine learning in CYANO-MLP appears robust to these sources of bias with high accuracy in phyloclassification of cyanobacterial genomes. CYANO-MLP consistently classifies plastids as late-branching Cyanobacteria, consistent with independent evidence from signature-based approaches and some previous phylogenetic studies.

## Background

Over one billion years ago [1–3] photosynthetic eukaryotes originated through endosymbiosis of a cyanobacterium with the last common ancestor of Archaeplastida, a eukaryotic supergroup encompassing green and red algae, land plants, and glaucophytes [4–6]. The diversity of eukaryotic photoautotrophs radiating from this event profoundly transformed the terrestrial biosphere through changes to primary biomass production, atmospheric oxygenation, and the colonization of new ecosystems [7, 8]. It is widely accepted that plastids originated in a single primary endosymbiotic event [9]. However, the phylogenetic root of plastids within Cyanobacteria remains controversial. Recent phylogenomic studies reach contradictory conclusions, with plastids branching either early [10–13] or late [1, 14–16] within Cyanobacteria with strong statistical support.

Phylogenetic inferences concerning plastid origin are complicated by large evolutionary distances that have accumulated over at least one billion years of vertical descent, by extreme genome reductions in plastids [17] and in some Cyanobacteria [18, 19], and by secondary and tertiary endosymbiotic acquisitions of plastids. Genome reduction alters the stationary nucleotide compositions of genomes and the amino acid compositions of the proteins they encode [20], thereby violating the assumptions and applicability of many evolutionary models [21–26]. In contrast, evolutionary evidence from more signature-based approaches based on binary characters such as the presence or absence of endosymbiotic gene transfers [27], eukaryotic glycogen and starch pathways [28, 29], and conserved indels [30] more consistently point toward a late-branching origin of plastids. Uncertainty in the phylogenetic root of plastids precludes better understanding of early stages in plastid evolution and their environmental and metabolic contexts.

In this study, we show first that recently published phylogenomic datasets previously assembled from cyanobacterial and plastid genomes to address the root of plastids poorly fit the evolutionary models and character recoding strategies applied to them, which may help explain why earlier studies have reached contradictory conclusions with strong support. Then we introduce our Cyanobacterial Multi-Layer Perceptron Phyloclassifier ("CYANO-MLP"), a machine learning algorithm for phylogenetic classification of cyanobacterial and plastid query genomes to one of eight cyanobacterial clades. To "phyloclassify" a query genome to its clade of origin, the input data vector of CYANO-MLP respectively scores the query tRNA gene complement against eight sub-clade-specific structure-function maps for tRNAs called function logos, and the Class-Informative Features (CIFs) they contain [31].

The bioinformatic estimation of tRNA CIFs applies Molecular Information Theory [32] to databases of tRNA gene complements within clades, quantifying the function-specific information of tRNA structural features as potentially informative to tRNA-interacting proteins across 22 subfunctional alternative classes of tRNAs (the 20 standard elongator isoacceptor classes, the initiator class, and a special class of isoleucylated elongator tRNA), all assuming the tRNA genes we analyze make products functional in protein synthesis [31, 33, 34]. tRNAs engaged in protein synthesis are constrained to structurally conform to “fit” for passage through the ribosome in translation, allowing exquisite structural correspondence to be assigned across different functional classes. Phylogenomic inference based on tRNA CIFs has three advantages over traditional phylogenetic markers. The first is circumventing the need to determine orthology and paralogy of genes within and between genomes, because the markers are defined by integrating sequence information of the pooled gene complement of clades as a whole. Second, despite the extreme genome reduction observed within plastids and some cyanobacterial genomes, they mostly maintain full tRNA gene complements; the functional repertoire of tRNA gene complements are nearly universally conserved over the Tree of Life, even in organelles. Third, as shown in earlier work, a tRNA-CIF-based alphaproteobacterial phyloclassifier exhibited strong and robust recall and accuracy of phyloclassification, despite convergent nonstationary base compositions of tRNA gene complements in alphaproteobacterial genomes, and likely horizontal transfers of tRNA genes and genes for tRNA-interacting proteins [35]. In the present work, we use CYANO-MLP to find with strong and robust statistical support that the phylogenetic root of plastids lies in a late-branching cyanobacterial clade containing marine and freshwater, unicellular, and nitrogen-fixing ecotypes, previously shown to share synapomorphic starch metabolic pathway traits with plastids [1, 28]. Furthermore, we show that our main result of the late-branching cyanobacterial phyloclassification of plastids is robust to the deletion of clades included in the phyloclassifier model, unbalanced sampling of genomes across clades, and to compositional heterogeneity in input tRNA gene data.

## Results

### Evolutionary Models Fit Cyanobacterial and Plastid Phylogenomic Data Poorly

**Fig. 1:**
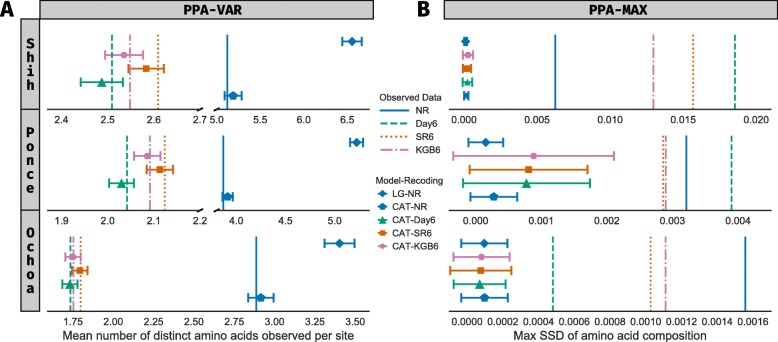
Results of posterior predictive analyses of phylogenomic datasets of Shih et al. [10], Ponce-Toledo et al. [12], and Ochoa de Alda et al. [14]. Rows within each panel correspond to phylogenomic datasets. Observed values calculated for each test statistic are represented by vertical lines. Color and patterns of vertical lines indicate amino acid recoding strategies, respectively NR: No recoding, DAY6: six-state Dayhoff recoding, SR6: the six-state recoding strategy of Susko and Roger [59], and KGB6: the six-state recoding strategy of Kosio et al. [60]. Symbols show average values for two posterior predictive test statistics calculated from simulated datasets, with error bars showing ± five standard deviations. Symbol shapes correspond to phylogenetic models (LG: LG+4 *Γ*, CAT: CAT-GTR+4 *Γ*) and symbol colors show recoding strategies. If similarly colored error bars contain vertical lines, the given phylogenetic model adequately describes systematic biases of the given data. **a** Results with the PPA-VAR statistic [22] assessing fit of models to site-process heterogeneity in the data. **b** Results with the PPA-MAX statistic, measured as squared standard deviation (SSD) of amino acid composition [23], showing generally poor fit of models against the lineage-specific compositional heterogeneity in the data

We used Posterior Predictive Analysis [22, 23] to examine the fit of recently used evolutionary models and data recoding strategies to three published cyanobacterial and plastid phylogenomic datasets (Fig. 1 and Additional file 1: Table S1). We found that the empirical matrix model with site-rate heterogeneity LG+4 *Γ* [36], which was applied to cyanobacterial and plastid data in Shih et al. [10], Ponce-Toledo et al. [12], and Ochoa de Alda et al.[14], fits site-process heterogeneity in all three phylogenomic datasets poorly (Fig. 1A and Additional file 1: Table S1). Previous work has shown that empirical matrix models fail to accommodate data with site-specific constraints that cause substitution process heterogeneity [37], and, when applied to such data, can bias phylogenetic estimation by long-branch attraction [22]. On the other hand, the CAT-GTR+4 *Γ* model [22, 37], which was applied to cyanobacterial and plastid data in Ponce-Toledo et al. [12] and Ochoa de Alda et al. [14], specifically accommodates heterogeneous substitution processes over sites in all three datasets (Fig. 1A and Additional file 1: Table S1). Yet, even when combined with amino-acid recoding intended to mitigate compositional heterogeneity as in Ponce-Toldeo et al. [12], the CAT-GTR+4 *Γ* model fits lineage-composition heterogeneity poorly in all three datasets (|*Z*|≥5, Fig. 1B and Additional file 1: Table S1). When lineage-composition heterogeneity is not adequately modeled, unrelated sequences with similar compositions may artifactually cluster together in reconstructed phylogenetic trees [23].

### Training and Validation of the CYANO-MLP Phyloclassifier

**Fig. 2:**
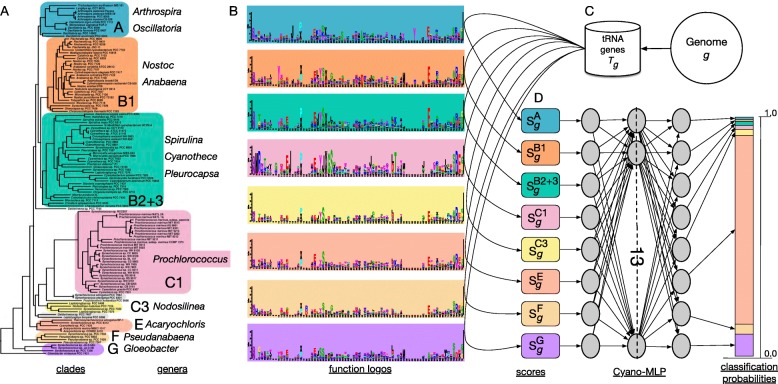
Schematic overview of CYANO-MLP phyloclassification workflow. **a** The cyanobacterial phylogeny from [10] was used to define cyanobacterial clades for which we estimated tRNA CIFs. Cyanobacterial clades, indicated by background colors, were named according to [10], except for clade B2+3, which combines clades B2 and B3. Clades with grey backgrounds were excluded from analysis because of limited genome sample sizes. Next, tRNA genes are predicted from cyanobacterial genomes and combined by clade for function logo estimation [31]. **b** Example function logos, for Uracil, with background colors corresponding to each cyanobacterial clade. **c** For a genome *g* to be classified, tRNA gene complements *T*_*g*_ are predicted and scored against the logos for each clade, to produce input score vectors for CYANO-MLP training and classifications. **d** The architecture of the artificial neural network CYANO-MLP, and a classification probability vector output from CYANO-MLP represented as a stacked bar chart

We annotated 5,476 tRNA genes in 117 cyanobacterial genomes analyzed in Shih et al. [10], averaging 46.80 tRNA genes per cyanobacterial genome. We also annotated and extracted 14,841 tRNA genes from 440 Archaeplastida plastid genomes averaging 33.73 tRNA genes per plastid genome, 44 tRNA genes from the cyanobacterium *Gloeomargarita lithophora*, and 42 tRNA genes from the chromatophore genome of the fresh-water amoeba *Paulinella chromatophora* (Table 1). Using the clade nomenclature of Shih et al. [10], we excluded clades C2 and D from further analysis (Fig. 2; grey clades) because of limited total tRNA gene numbers. We ultimately trained our CYANO-MLP phyloclassifier on tRNA CIFs estimated from 5,720 tRNA gene sequences in 113 genomes among cyanobacterial clades A, B1, B2+3, C1, C3, E, F, and G (Fig. 2, Additional File 2: Figures S1-S8, and Additional File 1: Tables S2,S3). We fused clades B2 and B3 because B3 contained only one genome, and they are sister clades [10, 13, 14].

**Table 1:** Summary statistics on genomes and tRNA genes of cyanobacterial and plastid clades and grades

Clade/Grade	Genomes (G)	Genes (T)	T/G	Bases (N)	N/T	%A	%T	%G	%C
Cyanobacteria									
A	11	555	50.45	40,362	72.72	20.1	23.3	31.2	25.4
B1	27	1,395	51.67	101,640	72.86	19.7	23.3	31.6	25.4
B2+3	30	1,314	43.80	95,685	72.82	19.5	23.0	32.0	25.5
C1	29	1,205	41.55	87,856	72.91	18.8	21.8	32.7	26.7
C2	2	90	45.00	6,550	72.78	19.5	21.9	32.2	26.4
C3	3	142	47.33	10,346	72.86	19.1	22.8	32.3	25.7
D	2	116	58.00	8,430	72.67	19.7	23.3	31.7	25.3
E	5	266	53.20	19,378	72.85	19.3	22.8	32.1	25.8
F	4	211	52.75	15,339	72.70	20.1	23.4	31.3	25.2
G	4	182	45.50	13,218	72.63	18.7	21.7	32.8	26.8
G. lithophora	1	44	44	3,194	72.59	18.8	22.7	32.3	26.2
Plastids/Chromatophores									
Charophyta	10	352	35.20	25,513	72.48	21.1	24.2	30.1	24.6
Chlorophyta	7	226	32.29	16,436	72.73	21.9	25.4	29.1	23.6
Cryptophyta	4	117	29.25	8,512	72.75	20.9	24.2	29.9	25.0
Heterokonta	33	992	30.06	72,229	72.81	21.2	25.1	29.6	24.1
Eudicots	191	6,593	34.52	478,337	72.55	21.4	25.2	29.7	23.7
Euglenaceae	10	276	27.60	20,070	72.72	22.3	27.1	28.4	22.3
Monilophytes	8	270	33.75	19,641	72.74	21.2	24.5	30.0	24.3
Gymnospermae	26	824	31.69	59,867	72.65	21.9	24.6	29.6	23.9
Haptophyta	4	111	27.75	8,079	72.78	21.0	25.1	29.7	24.2
Monocots	112	3930	35.10	285,302	72.60	21.7	25.2	29.5	23.7
Magnoliids	9	315	35.00	22,856	72.56	21.3	24.9	29.7	24.0
Nymphaeales	2	68	34.00	4,934	72.56	21.4	25.1	29.8	23.7
Rhodophyta	20	624	31.20	45,438	72.82	21.9	25.2	29.1	23.8
Bryophyta	3	108	36.00	7,845	72.64	22.2	25.4	29.0	23.4
Glaucocystophyta	1	35	35	2,543	72.66	20.4	23.7	30.9	25.0
*P. chromatophora*	1	42	42	3,060	72.86	19.1	21.7	32.7	26.5

**Fig. 3:**
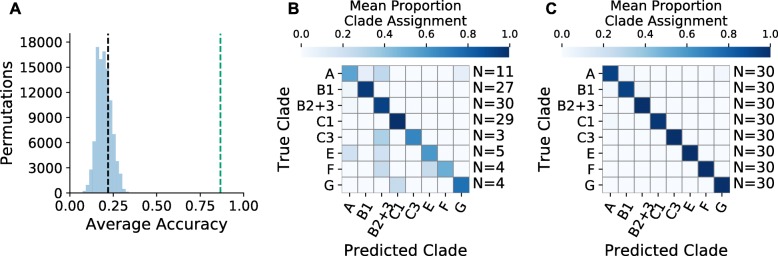
Training results of CYANO-MLP. **a** Null distribution of classification accuracy generated using 100,000 permutations of score vectors with clade labels swapped. The black line indicates expected accuracy if genomes were randomly assigned to clades and the green line indicates accuracy of CYANO-MLP using leave one out cross-validation. Normalized confusion matrices with the number of genomes used for training indicated on the right side for **b** CYANO-MLP and a **c** clade balanced version of CYANO-MLP produced by oversampling under-represented clades

We trained CYANO-MLP on input vectors that score query cyanobacterial genomic tRNA gene complements against CIFs estimated for separate cyanobacterial clades. We optimized the parameters and architecture of CYANO-MLP on the training data, settling on a single hidden layer of 13 nodes (Fig. 2), which achieved an average accuracy of 0.8673 (*p*=0.0001; Figs. 3A, 3B and Additional File 1: Tables S4,S5). Notably, misclassifications were concentrated among cyanobacterial clades with limited training data (Fig. 3B and Table 2) with Clade A receiving the lowest precision score, and the lowest recall and balanced accuracy score among late-branching clades (i.e. A, B1, B2+3) (Table 2). To examine the effects of unbalanced training data on the performance of CYANO-MLP, we created a separate clade-balanced version by oversampling data from under-represented clades. The synthetically clade-balanced version achieved an accuracy of 0.9875 with improvements in precision and recall for all clades (Fig. 3C and Table 3), demonstrating that these misclassifications are a result of biased taxonomic sampling and not an inability of CYANO-MLP to distinguish cyanobacterial clades. However, even with oversampling, clade A received the lowest precision, recall, and balanced accuracy scores among all clades, possibly because of undersampling relative to diversity specifically within clade A (Table 3). Furthermore, the phylogenetic signal across tRNA CIFs is consistent; cyanobacterial genomes were correctly classified in at least 97 of 100 tRNA CIF bootstrap replicates of CYANO-MLP. These results indicate that CYANO-MLP is robust to variability in the G+C content of tRNA genes in both Cyanobacteria and plastids/chromatophores, which ranges between 56.5% and 59.6% across cyanobacterial clades and between 50.7% and 59.2% in plastid clades and chromatophores (Table 1).

**Table 2:** One-vs-all calculations of Precision, Recall, and Balanced Accuracy for CYANO-MLP for each Cyanobacterial clade

Clade	Precision	Recall	Balanced Accuracy
A	0.6000	0.5455	0.7439
B1	0.8966	0.9630	0.9640
B2+3	0.8000	0.9333	0.9245
C1	1.0000	1.0000	1.0000
C3	1.0000	0.6667	0.8333
E	1.0000	0.4000	0.7000
F	1.0000	0.5000	0.7500
G	0.7500	0.7500	0.8704

Defined as $Precision = \frac {tp}{tp+fp}$, $Recall = \frac {tp}{tp+fn}$, $Balanced Accuracy = \frac {TPR+TNR}{2}$, where tp = true positive, fp = false positive, fn = false negative, TPR = true positve rate, and TNR = true negative rate

**Table 3:** One-vs-all calculations of Precision, Recall, and Balanced Accuracy for CYANO-MLP-BAL for each Cyanobacterial clade

Clade	Precision	Recall	Balanced Accuracy
A	0.9394	0.9300	0.9350
B1	0.9687	0.9300	0.9500
B2+3	0.9709	1.0000	0.9850
C1	1.0000	0.9700	0.9850
C3	1.0000	1.0000	1.0000
E	1.0000	1.0000	1.0000
F	1.0000	1.0000	1.0000
G	0.9709	1.0000	0.9850

Defined as $Precision = \frac {tp}{tp+fp}$, $Recall = \frac {tp}{tp+fn}$, $Balanced Accuracy = \frac {TPR+TNR}{2}$, where tp = true positive, fp = false positive, fn = false negative, TPR = true positve rate, and TNR = true negative rate

**Fig. 4:**
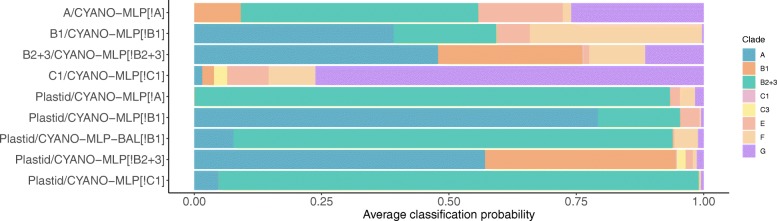
Average classification probabilities for genomes with clade data excluded from training. *Y*/CYANO-MLP[*!X*] indicates results for clade *Y* genomes classified with a version of CYANO-MLP with clade *X* data excluded from training, where *X* is either A, B1, B2+3, or C1. Results indicate that equivocal classification probabilities suggest a “none-of-the-above” classification

Given that the cyanobacterial clade from which plastids (or other queries) truly arose may not be included among clades represented in CYANO-MLP, we undertook to investigate the ability of CYANO-MLP to signal “none-of-the-above.” We optimized and trained variants of CYANO-MLP leaving out each one of clades A, B1, B2+3, or C1 and reclassifying all cyanobacterial genomes, including members of the clade that had been left out. Overall, accuracies were similar to those of CYANO-MLP for each delete-clade model variant (Additional file 2: Figure S9 and Additional file 1: Table S5). Consistently, phyloclassifications of cyanobacterial genomes from excluded clades were more equivocal than those of genomes from included clades, with genomes from excluded clades obtaining on average maximum classification probabilities less than 80% (Fig. 4 and Additional file 1: Tables S5,S6). Based on these results and criteria, we interpret equivocal classifications with CYANO-MLP as indicating “none-of-the-above.”

### The *Paulinella chromatophora* Chromatophore Phyloclassifies to the Marine C1 *Prochlorococcus* and *Synechococcus* Clade

**Fig. 5:**
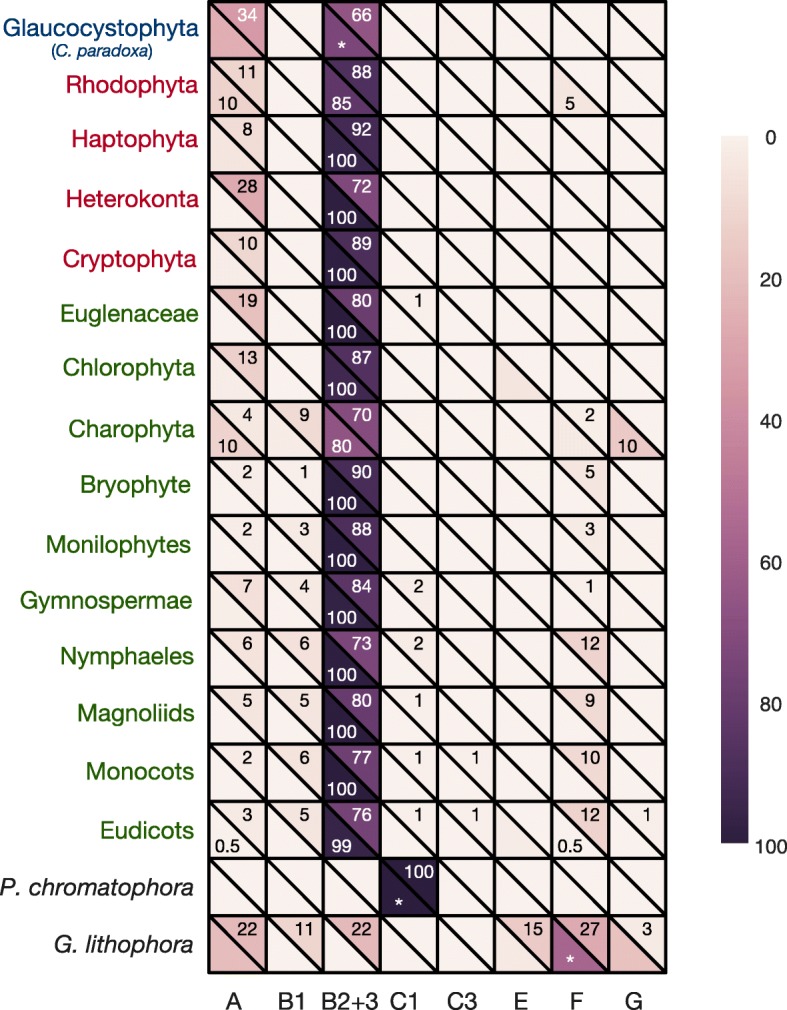
CYANO-MLP classification results for genomes of plastids, the chromatophore of *P. chromatophora*, and the cyanobacterium *G. lithophora*. Row label colors denote Archeaplastid clades with Rhodophyta in red, Chloroplastida in green, and Glaucocystophyta in blue, or non-Archaeplastida in black. Heatmap lower half-cells show average probabilities of classifications of genomes to clades with text labels denoting percentages of genomes classifying to cyanobacterial clades. Asterisks (*) denote single genomes. Heatmap upper half-cell colors (and text labels) show median bootstrap classification frequencies (as percentages). Absence of labels denotes zero frequencies

As organelles, plastid genomes experienced distinctive selection pressures that hypothetically could cause idiosyncratic score vectors and artifactual classification with CYANO-MLP. To investigate this possibility, we classified the chromatophore genome of the fresh-water amoeba *P. chromatophora*. The chromatophore is a photosynthetic organelle representing a second primary endosymbiosis event presumably under similar selection pressures as plastid genomes. The phylogenetic origin of the chromatophore from the marine *Prochlorococcus* and *Synechococcus* clade (clade C1; Fig. 2) is well-supported in several phylogenomic analyses [10, 13, 14]. CYANO-MLP classified the chromatophore concordantly to clade C1 with a 99.98% probability and 100% bootstrap support (Fig. 5, and Additional file 1: Table S7). Phyloclassification of the *P. chromatophora* chromatophore was robust to model re-specification and biased phylogenetic sampling, with similar results for delete-clade and oversampled clade-balanced models (Additional file 1: Table S5). In addition, the chromatophore classified similarly to other C1 cyanobacteria when using the C1 delete-clade model (Additional file 1: Table S5). Notably, the chromatophore was classified correctly despite clade C1 tRNA-interacting proteins following a complex evolutionary history including horizontal gene transfers and duplications [38].

### Plastids Phyloclassify as Late-Branching Cyanobacteria

Using CYANO-MLP, we obtained robust and consistent support for a late-branching origin of plastids within or closely related to the B2+3 clade of Cyanobacteria (Fig. 5 and Additional file 1: Tables S6,S7). CYANO-MLP phyloclassified 433 plastid genomes to the B2+3 clade and four plastid genomes to the A clade (Fig. 5; Additional file 1: Table S7), for a total of 437 of 440 (99.32%) plastid genomes classifying to late-branching clades with high probabilities. Among 433 plastid genomes classifying to B2+3, 408 (or 94.2%) scored against B2+3 with a probability of 98.5% or better (Additional file 1: Tables S8,S9). Genomes of all three major Archaeplastida lineages phyloclassified as late-branching B2+3 Cyanobacteria. Excluding Charophyta and Glaucocystophyta (11 genomes), the median classification probability of plastid clades against B2+3 Cyanobacteria was over 99%, except in the eudicots where it was over 97% (Additional file 1: Tables S8,S9). The maximum classification probability was over 99% for all plastid clades except Glaucocystophyta. Considering the low precision, recall, and balanced accuracy of clade A and low average classification probabilities of plastids to clade A (0.0137±0.0717), we interpreted the phyloclassifications of four plastid genomes to clade A as likely false positives.

The majority of plastid bootstrap replicates classified to late-branching clades A, B1, and B2+3, with the median bootstrap frequencies of all plastid groups at or above 70 against clade B2+3, except for the one Glaucocystophyta genome (Fig. 5 and Additional file 2: Figures S10,S11). Three plastid genomes classified to early-diverging cyanobacterial clades; two to clade F and one to clade G (Fig. 5 and Additional file 1: Tables S7,S8). Clade-balancing did not improve consistency, with 384 and 18 plastids phyloclassifying into clades B2+3 and A respectively (Additional file 1: Table S8), suggesting inherent limitations with current methods and data. However, plastid genome classifications were robust to model specification and the inclusion or exclusion of clades, with results largely unchanged using the delete-clade-A or delete-clade-C1 variants (Fig. 4, Additional file 2: Figure S12, and Additional file 1: Tables S5,S6,S8). Distinctly, plastid classifications with the delete-clade B1 variant were more equivocal between clades A and B2+3 (Fig. 4 and Additional file 1: Tables S5,S6,S8). However, after oversampling and retraining of the delete-clade B1 variant, phyloclassifications were similar to those with CYANO-MLP and clade-balanced CYANO-MLP (Fig. 4 and Additional file 1: Tables S5,S6,S8), suggesting that balanced sampling is a limiting factor in the robustness of phyloclassification to clade-deletion. Remarkably however, phyloclassifications of plastids and B2+3-cyanobacteria were mutually consistent and similarly equivocal, using the delete-clade-B2+3 model variant of CYANO-MLP (Fig. 4, Additional file 2: Figure S12, and Additional file 1: Table S7,S9). We interpret this as consistent with a common “none-of-the-above” phyloclassification for both groups using the delete-B2+3 clade version of CYANO-MLP. Overall, despite considerable heterogeneity in plastid gene and nucleotide compositions after at least a billion years of evolutionary divergence and limited and unbalanced taxonomic sampling in training data, CYANO-MLP phyloclassifications of plastid genomes as B2+3 Cyanobacteria are remarkably consistent and robust.

### *Gloeomargarita lithophora* Phyloclassifies as an Early-Branching Cyanobacteria

Recent phylogenomic analyses have supported a sister relationship between plastids and an early-diverging lineage containing *G. lithophora* as its only member [12, 13]. With only one genome, there was insufficient tRNA sequence data to estimate CIFs for this lineage. Instead, we classified the *G. lithophora* genome using CYANO-MLP to determine if it classified similarly to plastids, which would be consistent with a sister relationship of *G. lithophora* and plastids. We found that the *G. lithophora* genome obtained greater than 75% total classification probability against three early-diverging clades, classifying to clade F with probability 57.3%, to clade G with probability 18.4%, and to clade E with probability 3.2%. In addition, *G. lithophora* classified to the late-diverging clade A with probability 20.3% likely a result of the low precision, recall, and balanced accuracy of clade A (Fig. 5). We interpreted these results as consistent with a “none-of-the-above” classification, yet, favoring an early-branching of *G. lithophora*, in agreement with recent phylogenomic analyses [12, 13]. However, the incongruity of our results for *G. lithophora* and plastids rejects their sister relationship.

## Discussion

Using our phyloclassification approach, we recovered strong support for the phylogenetic root of plastids within or closely related to the B2+3 clade of Cyanobacteria (Figs. 2, 5 and Additional file 1: Tables S6-S9). Our result is robust to bootstrap-resampling of tRNA structural positions (Fig. 5 and Additional file 2: Figures S10-S11), model specification (Additional file 2: Figure S12 and Additional file 1: Table S6-S7), and unbalanced training data (Figs. 3, 5, Additional file 2: Figure S12, and Additional file 1: Tables S6-S9). Although our results are inconsistent with a sister relationship between plastids and the early-branching *G. lithophora* [12, 13] (Fig. 5), they are consistent with independent metabolic evidence that plastids originated from a unicellular starch-producing nitrogen-fixing cyanobacterial species [28, 29]. Notably, ancestral state reconstruction suggests that the common ancestor of the B2+3 clade lived in a freshwater habitat [8], supporting hypotheses that photosynthetic eukaryotes originated and diversified rapidly in a freshwater habitat [8, 13, 15].

Importantly, the significantly lower classification accuracy of CYANO-MLP on class-permuted training datasets (Fig. 3A) supports the interpretation that CYANO-MLP phyloclassifications depend on learned phylogenetic signals in cyanobacterial tRNA CIFs. Furthermore, both plastids and the *P. chromatophora* chromatophore classified consistently in multiple re-specifications of CYANO-MLP (Fig. 5, Additional file 2: Figures S10-12, and Additional file 1: Tables S6-9), arguing against the interpretation that our classifications of these genomes are artifacts of idiosyncratic evolutionary processes associated with the transition to becoming an organelle. Additionally, the similarly equivocal phyloclassifications of plastids and B2+3-cyanobacteria using the delete-clade-B2+3 model variant of CYANO-MLP (Fig. 4, Additional file 2: Figure S12, and Additional file 1: Table S7,S9) provides additional support that the progenitor of plastids was a cyanobacteria of the B2+3 clade or a close sister to it.

Early conflicting hypotheses of the phylogenetic position of plastids were possibly a consequence of limited sampling of cyanobacterial genomes and genes, but recent genome sequencing efforts have produced several large phylogenomic datasets that appear to fail to resolve whether plastids branch early or late within Cyanobacteria [1, 10–16, 39, 40]. When different phylogenomic datasets recover strongly supported, yet conflicting, hypotheses about evolutionary relationships, the reason is unlikely to be from lack of data, but rather poorly fitting phylogenetic models that are unable to adequately describe systematic variation in the data [23, 24, 41]. Our Posterior Predictive Analysis showed that current evolutionary models do not adequately fit cyanobacterial and plastid phylogenomic datasets because of site-process and lineage-composition heteogeneities. As expected, we found that the CAT-GTR+4 *Γ* model [22, 37] accommodated site-specific constraints (Fig. 1A and Additional file 1: Table S1), however, we show that amino acid recoding strategies did not completely mitigate lineage-specific compositional biases (Fig. 1B and Additional file 1: Table S1). Our results suggest that accurate reconstruction of the branching origin of plastids within Cyanobacteria requires a model that can accommodate both site-process and lineage-composition heterogeneities in the data to avoid biases in phylogenetic estimation. To our knowledge, only one previous phylogenomic study controlled both sources of bias, by both modeling 16S rDNA nucleotide data with the CAT-GTR+4 *Γ* model and removing compositionally divergent taxa [14]. Notably, their findings are consistent with ours in supporting a late-branching origin of plastids within Cyanobacteria [14].

## Conclusion

Common models of sequence evolution inadequately fit site-process and lineage-composition heteogeneities in cyanobacterial and plastid phylogenomic data sets. Phyloclassifications with CYANO-MLP, based on tRNA functional signatures, are accurate, robust and consistently and unambiguously support a late-branching origin of plastids, consistent with signature-based evidence from metabolic pathways[1, 28], endosymbiotic gene transfers [27], conserved gene indels [30], and phylogenomic approaches that account for both sources of systematic bias. Our machine learning approach holds promise to tackle other difficult phylogenomic problems.

## Methods

### tRNA Gene Data and Genome Sets

From NCBI, we downloaded the 117 cyanobacterial genomes analyzed in Shih et al. [10], the genome of the cyanobacterium *Gloeomargarita lithophora*, the genome of the chromatophore of the fresh-water amoeba *Paulinella chromatophora*, and 440 complete plastid genomes containing representatives from all three lineages of Archaeplastida (Glaucocystophyta, Rhodophyta, and Viridiplantae). For cyanobacterial and chromatophore genomes we annotated tRNA genes as the union of predictions from tRNAscan-SE v1.31 [42] in bacterial mode and ARAGORN v1.2.36 [43] with default settings. We annotated tRNA genes in plastid genomes similarly, except we discarded as false positives gene predictions from ARAGORN that contained introns in tRNA isotypes that have not been previously described to contain introns within plastid genomes [44–46]. We additionally filtered away tRNA gene predictions for land plant plastid genomes that contained anticodons not previously observed in land plant plastid tRNA genes [47, 48].

We annotated the functional types of tRNA genes either as elongator isotypes by anticodon alone or, for those containing the CAU anticodon, into initiator tRNA ^Met^ ("X"), elongator tRNA ^Met^, or tRNA ^Ile^_*CAU*_ ("J") using TFAM v1.4 [49] with the TFAM model used in [35, 50]. We aligned tRNA sequences using COVEA v2.4.4 [51] and the prokaryotic tRNA covariance model from tRNAscan-SE [42]. We edited the alignment by first removing sites containing 99% or more gaps using FAST v1.6 [52], and then removing sequences with unusual secondary structure. Lastly, we mapped sites to Sprinzl coordinates [53] and manually removed the variable arm, CCA tail, and sites not mapping to a Sprinzl coordinate using Seaview v4.6.1 [54]. The alignment is available at 10.6084/m9.figshare.8298662 and https://github.com/tlawrence3/CYANO-MLP.

We partitioned cyanobacterial tRNA genes into sets *T*_*g*_ for each genome *g* of origin, and separately into sets *T*_*X*_ for each cyanobacterial clade *X*, with *X*∈*CC*≡{A,B1,B2+3,C1,C3,E,F,G} corresponding to clades identified in [10], except for fusion of clades B2 and B3 into their union B2+3 and exclusion of four genomes in two clades, C2 and D, for insufficient data as defined by yielding fewer than 120 tRNA genes (Fig. 1). Let *R*⊂*S* be the set of all 113 cyanobacterial genomes not excluded. For every cyanobacterial genome *g*∈*R* and for each cyanobacterial clade *X*∈*CC*, we also created leave-one-out cross-validation training sets $T_{X}^{g} = T_{X} - T_{g}$, by removing the tRNA genes of genome *g* from set of tRNA genes included in clade *X*.

### Genome Scoring

Following Amrine et al. [35], we produced training input vectors by first calculating clade-dependent Gorodkin heights [35, 55] $h^{i}_{f,X}$, in function logos [31] to estimate CIFS for each of eight cyanobacterial clade-specific tRNA gene sets, *T*_*X*_, and for each leave-one-out cross-validation training sets, $T_{X}^{g}$, with *X*∈*CC*, for all features *f*∈*F*≡{*A*,*C*,*G*,*U*}×*SC*, where *SC* is the set of Sprinzl Coordinates [53], and for all functional types *i*∈*I*≡*A*∪{J,X}, where *A* is the set of short IUPAC amino acid symbols standing for aminoacylation identities of elongators. We performed the calculations to estimate function logos using custom software tSFM available at https://github.com/tlawrence3/tsfm/tree/v0.9.6.

To score the tRNA gene complement *T*_*g*_ of genome *g*, we calculated a vector of tRNA CIF-based scores $\mathbf {S_{g}} = \langle S_{g}^{A},S_{g}^{B1},S_{g}^{B2+3},S_{g}^{C1},S_{g}^{C3},S_{g}^{E},S_{g}^{F},S_{g}^{G}\rangle $, in which element $S_{g}^{X}$ is the average, over all genes *t*∈*T*_*g*_ of any type *i*_*t*_∈*I*, where *i*_*t*_ is the type of gene *t*, of the sum over all features *f*∈*t*⊂*F* contained in that gene, of the Gorodkin heights [55] of those features for genes of that type in clade *X*∈*CC* calculated from the leave-one-out cross-validation data set of genome *g*:

1 $$ S_{g}^{X} \equiv \frac{1}{\vert T_{g} \vert}\sum_{t\in T_{g}}\sum_{f \in t} h^{i_{t}}_{f,X},   $$

A script for calculating score vectors from a set of tRNA sequences against a set of function logos is available at 10.6084/m9.figshare.8298662 and https://github.com/tlawrence3/CYANO-MLP.

Following recommended practice [56], we standardized score vectors of both training and query data by subtracting the mean score vector of training data and dividing element-wise by the standard deviations of scores by clade. Let $\mathbf {S_{g}^{\prime }}$ be the standardized score vector of *S*_*g*_.

### Phyloclassifier Model Training and Optimization

We implemented our multilayer neural network phyloclassifier using the MLPClassifier API of scikit-learn v0.18.1 [57] in Python v3.5.2. We trained models for up to 2000 training epochs, stopping early if for two consecutive iterations the Cross-Entropy loss function value did not decrease by a minimum of 1×10^−4^, and with random shuffling of data between epochs. We used the rectifier activation function for hidden layer neurons, the L-BFGS algorithm for weight optimization, and an alpha value of 0.01 for the L2 regularization penalty parameter. Lastly, we used the soft-max function to calculate classification probability vectors. Using leave-one-out cross-validation (LOOCV), we optimized neural network architecture for accuracy averaged over genomes *g*∈*R* considering all architectures with from one to four hidden layers and each layer individually containing from eight to sixteen nodes. To test the statistical significance of the average accuracy from LOOCV of the architecture-optimized CYANO-MLP, we permuted clade labels over training data in 100,000 replicates, followed by LOOCV and model retraining for each replicate.

### Phyloclassification and Bootstrapping

For each genomic tRNA gene set *T*_*g*_, for plastid, *P. chromatophora*, and *G. lithophora genomes*, we computed a standardized score vector $\mathbf {S_{g}^{\prime }}$, input this to CYANO-MLP, and classified to the clade with largest classification probability. To examine the consistency of phylogenetic signals in our data, we computed 100 bootstrap replicates of sites in our alignment of training and test tRNA gene data, followed by CIF-estimation, model retraining, and genome scoring and classification with each bootstrap replicate of CYANO-MLP. We summarized bootstrap results for cyanobacterial genomes by the number of replicates in which the most probable classification for a genome was its true clade of origin.

### Leave-Clade-Out and Balanced Model Variants

To examine the sensitivity of CYANO-MLP to missing data and model mis-specification, we re-optimized and re-trained models after leaving out either cyanobacterial clade A, B1, B2+3, or C1. To produce clade-balanced training datasets, we randomly resampled training score vectors so that each cyanobacterial clade had sample sizes equal to the best-sampled clade, and then re-optimized and re-trained models.

### Evaluation of Phylogenetic Model Adequacy

We examined goodness of fits of the phylogenomic datasets of Shih et al. [10], Ponce-Toledo et al. [12] (chloroplast-marker dataset) and Ochoa de Alda et al. [14](dataset 11) with the substitution models originally used in those studies, namely LG+4 *Γ* [36] and CAT-GTR+4 *Γ* [22, 37]. Posterior Predictive Analyses (PPA) were performed to test fits for site-specific constraint biases using PPA-DIV [22] and across-lineage compositional biases using PPA-MAX and PPA-MEAN [23]. Additionally, we assessed model adequacy under three amino acid recoding strategies, Dayhoff-6 (Day6) [58], the six-state recoding strategy of Susko and Roger [59] (SR6), and the six-state recoding strategy of Kosiol et al. [60] (KGB6). All PPA test statistics were calculated using at least 1000 samples from the posterior distribution after discarding burn-in. PPA results were interpreted using Z-scores under the assumption that the test statistics follow a normal distribution. We used a Z-score threshold of *Z*≥|5| as strong evidence for rejecting the model. We performed phylogenetic analyses using Phylobayes MPI v1.8 [61, 62] running two MCMC chains in parallel for each analysis. Convergence of chain trajectories was assessed using TRACECOMP and BPCOMP utilities provided with Phylobayes MPI. Convergence was assumed when the discrepancies of model parameters and bipartition frequencies between independent chains was less than 0.18. The number of cycles to discard as burn-in was determined by visually examining the traces of the log-likelihood and other model parameters for stationarity using Tracer v1.6.0.

## Supplementary information

**Additional file 1** **Table S1** Results of the posterior predictive analyses presented as z-scores. **Table S2** Descriptive statistics of Cyanobacterial clade function logos. (Stack Height/Symbol) Average of information content for each site divided by the number of symbols for that site, (Symbols) Average number of symbols per site, and (Stack Height) the average information content in bits of each site. Sites with zero information were excluded from calculations. **Table S3** Information content per nucleotide for each Cyanobacterial clade measured in bits. Number in parenthesis is percent of total information. **Table S4** Network architecture and average accuracy using Leave-One-Out Cross-Validation for all model variants of CYANO-MLP. Where CYANO-MLP[!*X*] indicates variant of CYANO-MLP with clade *X* data excluded from training and BAL indicates clade balanced training data. **Table S5** Mean probability plus and minus one standard deviation of classification for the indicated group (Grp. Class.) using the specified variant of CYANO-MLP. Where CYANO-MLP[!*X*] indicates variant of CYANO-MLP with clade *X* data excluded from training and BAL indicates clade balanced training data. **Table S6** Number of plastid genomes and left-out cyanobacterial clade genomes classifying to each cyanobacterial clade for the indicated version of CYANO-MLP. The number outside of parentheses indicated number of plastid genomes and the number within parentheses is the left-out cyanobacterial clade genomes. Dashes indicate N/A values. [!*X*] indicates variant of CYANO-MLP with clade *X* data excluded from training and BAL indicates clade balanced training data. **Table S7** Classification results for plastid genomes and the chromatophore of *P. chromatophora* using CYANO-MLP. Results are summarized by plastid groups. Number of genomes classifying to each Cyanobacterial clade and percent are shown. 408 out of the 433 plastid genomes scored against B2+3 with a probability of 98.5% or better. **Table S8** Number of plastid genomes with indicated max classification probability with the indicated version of CYANO-MLP. Where CYANO-MLP[!*X*] indicates variant of CYANO-MLP with clade *X* data excluded from training and BAL indicates clade balanced training data. **Table S9** Median, mean, and standard deviation of classification probability to Cyanobacteria clade B2+3 for plastid genome groups.

**Additional file 2** **Figure S1** Function Logos for Cyanobacterial Clade A. **Figure S2** Function Logos for Cyanobacterial Clade B1. **Figure S3** Function Logos for Cyanobacterial Clade B2+3. **Figure S4** Function Logos for Cyanobacterial Clade C1. **Figure S5** Function Logos for Cyanobacterial Clade C3. **Figure S6** Function Logos for Cyanobacterial Clade E. **Figure S7** Function Logos for Cyanobacterial Clade F. **Figure S8** Function Logos for Cyanobacterial Clade G. **Figure S9** Normalized confusion matrices for **(A)** CYANO-MLP[!A], **(B)** CYANO-MLP[!B1], **(C)** CYANO-MLP[!B2+3], **(D)** CYANO-MLP[!C1], and **(E)** CYANO-MLP-BAL[!B1]. Where CYANO-MLP[!*X*] indicates variant of CYANO-MLP with clade *X* data excluded from training and BAL indicates clade balanced training data. **Figure S10** Classification results of 100 bootstrap replicates of each Rhodophyta derived plastid genome. Results are summarized by Red plastid group with boxes spanning from the 25th percentile (bottom) to the 75th percentile (top) of bootstrap replicates classifying to the indicated Cyanobacterial clade per genome with the bisecting line marking the median value. Error bars indicate the shorter of either ± the interquartile range or the span of bootstrap replicates per genome. Dots show bootstrap replicates for individual genomes. Plastid genome bootstrap replicates classifying to cyanobacteral clades **(A)** A, B1, B2+3, **(B)** C1, C3, E, F, or G. **Figure S11** Classification results of 100 bootstrap replicates of each Chloroplastida derived plastid genome. **(A)** Bootstrap results for plastid genomes classifying to cyanobacterial clades A, B1, B2+3, C1, F, and G. **(B)** Bootstrap results for plastid genomes classifying to cyanobacterial clades C3 and E. **Figure S12** Box plot of maximum classification probability of each plastid genome. Error bars are the lesser of 1.5 IQR or full range of data. CYANO-MLP[!*X*] indicates variant of CYANO-MLP with clade *X* data excluded from training and BAL indicates clade balanced training data.

## Data Availability

The datasets generated during and/or analysed during the current study are available in the figshare repository 10.6084/m9.figshare.8298662.[63].

## Abbreviations

CIF: Class informative feature
Day6: Dayhoff 6-state recoding
KGB6: 6-state recoding strategy of Kosiol et al. (2004)
LOOCV: Leave-one-out cross-validation
MLP: Multilayer perceptron
NCBI: National center for biotechnology information
PPA: Posterior predictive analysis
SR6: 6-state recoding strategy of Susko and Roger (2007)
tRNA: Transfer ribonucleic acid
