# Supplementary material for: tRNA functional signatures classify plastids as late-branching cyanobacteria
Source: BMC Evol Biol. 2019 Dec 9;19:224. doi: 10.1186/s12862-019-1552-7 (PMC6902448; doi:10.1186/s12862-019-1552-7)
Supplement: Supplementary file 1 — Additional file 1 Table S1 Results of the posterior predictive analyses presented as z-scores. Table S2 Descriptive statistics of Cyanobacterial clade function logos. (Stack Height/Symbol) Average of information content for each site divided by the number of symbols for that site, (Symbols) Average number of symbols per site, and (Stack Height) the average information content in bits of each site. Sites with zero information were excluded from calculations. Table S3 Information content per nucleotide for each Cyanobacterial clade measured in bits. Number in parenthesis is percent of total information. Table S4 Network architecture and average accuracy using Leave-One-Out Cross-Validation for all model variants of CYANO-MLP. Where CYANO-MLP[!X] indicates variant of CYANO-MLP with clade X data excluded from training and BAL indicates clade balanced training data. Table S5 Mean probability plus and minus one standard deviation of classification for the indicated group (Grp. Class.) using the specified variant of CYANO-MLP. Where CYANO-MLP[!X] indicates variant of CYANO-MLP with clade X data excluded from training and BAL indicates clade balanced training data. Table S6 Number of plastid genomes and left-out cyanobacterial clade genomes classifying to each cyanobacterial clade for the indicated version of CYANO-MLP. The number outside of parentheses indicated number of plastid genomes and the number within parentheses is the left-out cyanobacterial clade genomes. Dashes indicate N/A values. [!X] indicates variant of CYANO-MLP with clade X data excluded from training and BAL indicates clade balanced training data. Table S7 Classification results for plastid genomes and the chromatophore of P. chromatophora using CYANO-MLP. Results are summarized by plastid groups. Number of genomes classifying to each Cyanobacterial clade and percent are shown. 408 out of the 433 plastid genomes scored against B2+3 with a probability of 98.5% or better. Table S8 Number of plastid genomes [file 12862_2019_1552_MOESM1_ESM.pdf]

Table S1: Results of the posterior predictive analyses presented as z-scores.

Data	Model	Recoding	PPA-Div	PPA-MAX	PPA-Mean
Shih	LG+4G	None	64.3264	308.247	112.4
	CAT-GTR+4G	None	3.48296	199.277	125.469
	CAT-GTR+4G	Dayhoff6	-2.41113	276.471	76.2146
	CAT-GTR+4G	KGB6	-1.54966	183.227	75.4934
	CAT-GTR+4G	SR6	-3.4146	272.776	75.4335
Ponce	LG+4G	None	102.301	57.7857	178.858
	CAT-GTR+4G	None	4.10787	41.3374	142.932
	CAT-GTR+4G	Dayhoff6	-2.18521	16.1799	25.4514
	CAT-GTR+4G	KGB6	-1.01998	8.22212	29.0685
	CAT-GTR+4G	SR6	-1.86252	11.4145	27.5351
Ochoa D11	LG+4G	None	27.8436	56.6042	49.6017
	CAT-GTR+4G	None	1.78031	56.2285	55.5332
	CAT-GTR+4G	Dayhoff6	-0.391998	14.0614	13.6587
	CAT-GTR+4G	KGB6	-0.242077	32.7976	20.3679
	CAT-GTR+4G	SR6	-0.449035	27.7049	16.1347

Table S2: Descriptive statistics of Cyanobacterial clade function logos. (Stack Height/Symbol) Average of information content for each site divided by the number of symbols for that site, (Symbols) Average number of symbols per site, and (Stack Height) the average information content in bits of each site. Sites with zero information were excluded from calculations.

Clade	Stack Height/Symbol	Symbols	Stack Height
A	0.22 ± 0.42	12.07 ± 6.12	1.02 ± 0.69
B1	0.17 ± 0.25	13.14 ± 6.24	1.12 ± 0.77
B2+3	0.21 ± 0.38	13.00 ± 6.18	1.25 ± 0.89
C1	0.51 ± 0.79	8.93 ± 5.67	1.63 ± 1.04
C3	0.26 ± 0.44	8.60 ± 5.09	0.85 ± 0.57
E	0.22 ± 0.33	9.53 ± 5.55	0.92 ± 0.59
F	0.17 ± 0.27	10.73 ± 5.69	0.78 ± 0.58
G	0.28 ± 0.49	8.99 ± 5.34	0.92 ± 0.62

Table S3: Information content per nucleotide for each Cyanobacterial clade measured in bits. Number in parenthesis is percent of total information.

Clade	A	C	G	U	Total Info
A	71.53(28.16)	55.13(21.70)	60.05(23.64)	67.32(26.50)	254.03
B1	87.85(29.77)	63.22(21.42)	65.98(22.36)	78.05(26.45)	295.10
B2+3	91.68(29.67)	67.92(21.98)	68.61(22.20)	80.84(26.16)	309.06
C1	114.57(29.20)	85.39(21.76)	78.72(20.06)	113.74(28.98)	392.42
C3	51.45(28.75)	35.27(19.71)	43.86(24.51)	48.37(27.03)	178.95
E	65.78(29.57)	46.40(20.85)	51.12(22.98)	59.18(26.60)	222.48
F	49.28(28.27)	35.91(20.60)	40.58(23.28)	48.53(27.84)	174.31
G	63.09(30.75)	39.22(19.12)	45.42(22.14)	57.43(27.99)	205.17

Table S4: Network architecture and average accuracy using Leave-One-Out Cross-Validation for all model variants of CYANO-MLP. Where CYANO-MLP[!*X*] indicates variant of CYANO-MLP with clade *X* data excluded from training and BAL indicates clade balanced training data.

Network	Classifier	LOOCV
(13)	CYANO-MLP	0.8673
(13, 15, 11, 13)	CYANO-MLP[!A]	0.9216
(13,15,13)	CYANO-MLP[!B1]	0.8721
(9, 13, 16, 10)	CYANO-MLP-BAL[!B1]	0.9952
(11, 12, 7, 10)	CYANO-MLP[!B2+3]	0.8795
(11, 9)	CYANO-MLP[!C1]	0.8214
(11, 14)	CYANO-MLP-BAL	0.9875

Table S5: Mean probability plus and minus one standard deviation of classification for the indicated group (Grp. Class.) using the specified variant of CYANO-MLP. Where CYANO-MLP[!X] indicates variant of CYANO-MLP with clade X data excluded from training and BAL indicates clade balanced training data.

Model Variant	Grp. Class.	A	B1	B2+3	C1	C3	E	F	G
CYANO-MLP	B2+3	0.0007 ± 0.0017	0.0003 ± 0.0005	0.9981 ± 0.0027	0.0000 ± 0.0000	0.0000 ± 0.0001	0.0002 ± 0.0007	0.0004 ± 0.0012	0.0001 ± 0.0003
CYANO-MLP	Plastids	0.0137 ± 0.0717	0.0007 ± 0.0102	0.9616 ± 0.1172	0.0000 ± 0.0010	0.0005 ± 0.0014	0.0132 ± 0.0275	0.0044 ± 0.0524	0.0058 ± 0.0395
CYANO-MLP[!A]	A	-	0.0915 ± 0.3009	0.4663 ± 0.4526	0.0006 ± 0.0019	0.0001 ± 0.0003	0.1654 ± 0.2724	0.0166 ± 0.0301	0.2596 ± 0.3886
CYANO-MLP[!A]	Plastid	-	0.0009 ± 0.0180	0.9335 ± 0.2379	0.0000 ± 0.0000	0.0000 ± 0.0005	0.0195 ± 0.1119	0.0293 ± 0.1563	0.0169 ± 0.1093
CYANO-MLP[!B1]	B1	0.3918 ± 0.4577	-	0.2015 ± 0.3671	0.0000 ± 0.0000	0.0008 ± 0.0020	0.0650 ± 0.2088	0.3379 ± 0.4463	0.0030 ± 0.0058
CYANO-MLP-BAL[!B1]	B1	0.3085 ± 0.4256	-	0.4386 ± 0.4759	0.0000 ± 0.0000	0.0000 ± 0.0001	0.0646 ± 0.2011	0.0635 ± 0.2357	0.1249 ± 0.3062
CYANO-MLP[!B1]	Plastid	0.7925 ± 0.3663	-	0.1612 ± 0.3312	0.0009 ± 0.0128	0.0000 ± 0.0002	0.0377 ± 0.1611	0.0027 ± 0.0349	0.0049 ± 0.0368
CYANO-MLP-BAL[!B1]	Plastid	0.0770 ± 0.2439	-	0.8622 ± 0.3159	0.0000 ± 0.0000	0.0000 ± 0.0002	0.0034 ± 0.0502	0.0465 ± 0.1742	0.0108 ± 0.0968
CYANO-MLP[!B2+3]	B2+3	0.4786 ± 0.4250	0.2831 ± 0.3924	-	0.0017 ± 0.0046	0.0002 ± 0.0002	0.0120 ± 0.0537	0.1097 ± 0.2544	0.1147 ± 0.2695
CYANO-MLP[!B2+3]	Plastid	0.5709 ± 0.4259	0.3743 ± 0.4194	-	0.0023 ± 0.0115	0.0174 ± 0.0774	0.0140 ± 0.0853	0.0078 ± 0.0584	0.0134 ± 0.0876
CYANO-MLP[!C1]	C1	0.0160 ± 0.0558	0.0229 ± 0.1193	0.0002 ± 0.0005	-	0.0259 ± 0.0350	0.0813 ± 0.1950	0.0916 ± 0.2202	0.7620 ± 0.3115
CYANO-MLP[!C1]	*P. chromatophora*	0.1244	0.0729	0.0001	-	0.2040	0.0027	0.1101	0.4858
CYANO-MLP[!C1]	Plastid	0.0474 ± 0.1969	0.0001 ± 0.0012	0.9431 ± 0.2190	-	0.0004 ± 0.0039	0.0005 ± 0.0072	0.0034 ± 0.0453	0.0051 ± 0.0653
CYANO-MLP-BAL	Plastids	0.0536 ± 0.1873	0.0038 ± 0.0179	0.8383 ± 0.3133	0.0005 ± 0.0014	0.0009 ± 0.0017	0.0192 ± 0.0605	0.0032 ± 0.0457	0.0804 ± 0.2320
CYANO-MLP-BAL	*P. chromatophora*	0.0000	0.0000	0.0000	.9999	0.0000	0.0000	0.0000	0.0000
CYANO-MLP-BAL	*G. lithophora*	0.0013	0.0021	0.0004	0.0004	.0001	0.0002	0.0149	0.9806

Table S6: Number of plastid genomes and left-out cyanobacterial clade genomes classifying to each cyanobacterial clade for the indicated version of CYANO-MLP. The number outside of parentheses indicated number of plastid genomes and the number within parentheses is the left-out cyanobacterial clade genomes. Dashes indicate N/A values. [!*X*] indicates variant of CYANO-MLP with clade *X* data excluded from training and BAL indicates clade balanced training data.

CYANO-MLP Version	A	B1	B2+3	C1	C3	E	F	G
[!B2+3]	263 (15)	165(8)	-(-)	0(0)	3(0)	3(0)	2(3)	4(4)
[!A]	-(-)	0(1)	412(5)	0(0)	0(0)	8(3)	14(0)	6(2)
[!C1]	19(0)	0(1)	417(0)	-(-)	0(0)	0(2)	2(2)	2(24)
[!B1]	354(11)	-(-)	67(5)	0(0)	0(0)	17(2)	1(9)	1(0)
BAL	18(-)	0(-)	384(-)	0(-)	0(-)	2(-)	1(-)	35(-)
BAL[!B1]	36(9)	-(-)	382(13)	0(0)	0(0)	1(3)	17(2)	4(3)

Table S7: Classification results for plastid genomes and the chromatophore of *P. chromatophora* using CYANO-MLP. Results are summarized by plastid groups. Number of genomes classifying to each Cyanobacterial clade and percent are shown. 408 out of the 433 plastid genomes scored against B2+3 with a probability of 98.5% or better.

Plastid Clade	A	B1	B2+3	C1	C3	E	F	G
Chlorophyta	0 (0%)	0 (0%)	7 (100%)	0 (0%)	0 (0%)	0 (0%)	0 (0%)	0 (0%)
Charophyta	1 (10%)	0 (0%)	8 (80%)	0 (0%)	0 (0%)	0 (0%)	0 (0%)	1 (10%)
Cryptophyta	0 (0%)	0 (0%)	4 (100%)	0 (0%)	0 (0%)	0 (0%)	0 (0%)	0 (0%)
Heterokonta	0 (0%)	0 (0%)	33 (100%)	0 (0%)	0 (0%)	0 (0%)	0 (0%)	0 (0%)
Eudicots	1 (0.52%)	0 (0%)	189 (98.95%)	0 (0%)	0 (0%)	0 (0%)	1 (0.52%)	0 (0%)
Euglenaceae	0 (0%)	0 (0%)	10 (100%)	0 (0%)	0 (0%)	0 (0%)	0 (0%)	0 (0%)
Monilophytes	0 (0%)	0 (0%)	8 (100%)	0 (0%)	0 (0%)	0 (0%)	0 (0%)	0 (0%)
Gymnospermae	0 (0%)	0 (0%)	26 (100%)	0 (0%)	0 (0%)	0 (0%)	0 (0%)	0 (0%)
Haptophyte	0 (0%)	0 (0%)	4 (100%)	0 (0%)	0 (0%)	0 (0%)	0 (0%)	0 (0%)
Monocots	0 (0%)	0 (0%)	112 (100%)	0 (0%)	0 (0%)	0 (0%)	0 (0%)	0 (0%)
Magnoliids	0 (0%)	0 (0%)	9 (100%)	0 (0%)	0 (0%)	0 (0%)	0 (0%)	0 (0%)
Nymphaeales	0 (0%)	0 (0%)	2 (100%)	0 (0%)	0 (0%)	0 (0%)	0 (0%)	0 (0%)
Rhodophyta	2 (10%)	0 (0%)	17 (85%)	0 (0%)	0 (0%)	0 (0%)	1 (5%)	0 (0%)
Bryophyte	0 (0%)	0 (0%)	3 (100%)	0 (0%)	0 (0%)	0 (0%)	0 (0%)	0 (0%)
Glaucocystophyta	0 (0%)	0 (0%)	1 (100%)	0 (0%)	0 (0%)	0 (0%)	0 (0%)	0 (0%)
Plastid total	4(0.91%)	0 (0%)	433 (98.41%)	0 (0%)	0 (0%)	0 (0%)	2 (0.45%)	1 (0.23%)
P. chromatophora	0 (0%)	0 (0%)	0 (0%)	1 (100%)	0 (0%)	0 (0%)	0 (0%)	0 (0%)

Table S8: Number of plastid genomes with indicated max classification probability with the indicated version of CYANO-MLP. Where CYANO-MLP[!*X*] indicates variant of CYANO-MLP with clade *X* data excluded from training and BAL indicates clade balanced training data.

Max Class. Prob.	CYANO-MLP	[!B2+3]	[!A]	[!C1]	[!B1]	BAL	BAL[!B1]
>=0.99	251	98	395	405	275	200	347
<0.99..>=0.95	121	166	24	15	75	136	37
<0.95..>=0.90	42	39	4	5	31	35	12
<0.90..>=0.85	8	19	1	4	9	19	6
<0.85..>=0.80	2	29	2	3	7	6	10
<0.80..>=0.75	4	14	1	3	11	9	8
<0.75..>=0.70	2	18	1	1	6	5	1
<0.70..>=0.65	3	13	3	1	8	4	3
<0.65..>=0.60	1	21	4	0	7	7	6
<0.60..>=0.55	2	8	0	1	2	4	4
<0.55..>=0.50	3	14	5	1	4	5	4
<0.50	1	1	0	1	5	10	2

Table S9: Median, mean, and standard deviation of classification probability to Cyanobacteria clade B2+3 for plastid genome groups.

Group	Median	Mean	Standard Deviation
Rhodophyta	0.9997	0.8259	0.3070
Nymphaeles	0.9955	0.9955	0.0018
Eudicots	0.9741	0.9590	0.0736
Monocots	0.9989	0.9980	0.0059
Gymnospermae	0.9947	0.9786	0.0458
Heterokonta	0.9977	0.9933	0.0114
Charophyta	0.7506	0.6615	0.3548
Monilophytes	0.9998	0.9855	0.0313
Bryophyte	1.0000	0.9956	0.0062
Haptophyta	0.9975	0.9460	0.0907
Cholorophyta	0.9996	0.9310	0.1670
Euglenaceae	0.9998	0.9987	0.0031
Magnoliids	0.9981	0.9977	0.0022
Glaucocystophyta	0.7330	0.7330	0.0000
Cryptophyta	0.9970	0.9922	0.0103
