# Supplementary material for: tRNA functional signatures classify plastids as late-branching cyanobacteria
Source: BMC Evol Biol. 2019 Dec 9;19:224. doi: 10.1186/s12862-019-1552-7 (PMC6902448; doi:10.1186/s12862-019-1552-7)
Supplement: Supplementary file 2 — Additional file 2 Figure S1 Function Logos for Cyanobacterial Clade A. Figure S2 Function Logos for Cyanobacterial Clade B1. Figure S3 Function Logos for Cyanobacterial Clade B2+3. Figure S4 Function Logos for Cyanobacterial Clade C1. Figure S5 Function Logos for Cyanobacterial Clade C3. Figure S6 Function Logos for Cyanobacterial Clade E. Figure S7 Function Logos for Cyanobacterial Clade F. Figure S8 Function Logos for Cyanobacterial Clade G. Figure S9 Normalized confusion matrices for (A) CYANO-MLP[!A], (B) CYANO-MLP[!B1], (C) CYANO-MLP[!B2+3], (D) CYANO-MLP[!C1], and (E) CYANO-MLP-BAL[!B1]. Where CYANO-MLP[!X] indicates variant of CYANO-MLP with clade X data excluded from training and BAL indicates clade balanced training data. Figure S10 Classification results of 100 bootstrap replicates of each Rhodophyta derived plastid genome. Results are summarized by Red plastid group with boxes spanning from the 25th percentile (bottom) to the 75th percentile (top) of bootstrap replicates classifying to the indicated Cyanobacterial clade per genome with the bisecting line marking the median value. Error bars indicate the shorter of either ± the interquartile range or the span of bootstrap replicates per genome. Dots show bootstrap replicates for individual genomes. Plastid genome bootstrap replicates classifying to cyanobacteral clades (A) A, B1, B2+3, (B) C1, C3, E, F, or G. Figure S11 Classification results of 100 bootstrap replicates of each Chloroplastida derived plastid genome. (A) Bootstrap results for plastid genomes classifying to cyanobacterial clades A, B1, B2+3, C1, F, and G. (B) Bootstrap results for plastid genomes classifying to cyanobacterial clades C3 and E. Figure S12 Box plot of maximum classification probability of each plastid genome. Error bars are the lesser of 1.5 IQR or full range of data. CYANO-MLP[!X] indicates variant of CYANO-MLP with clade X data excluded from training and BAL indicates clade balanced training data. [file 12862_2019_1552_MOESM2_ESM.pdf]

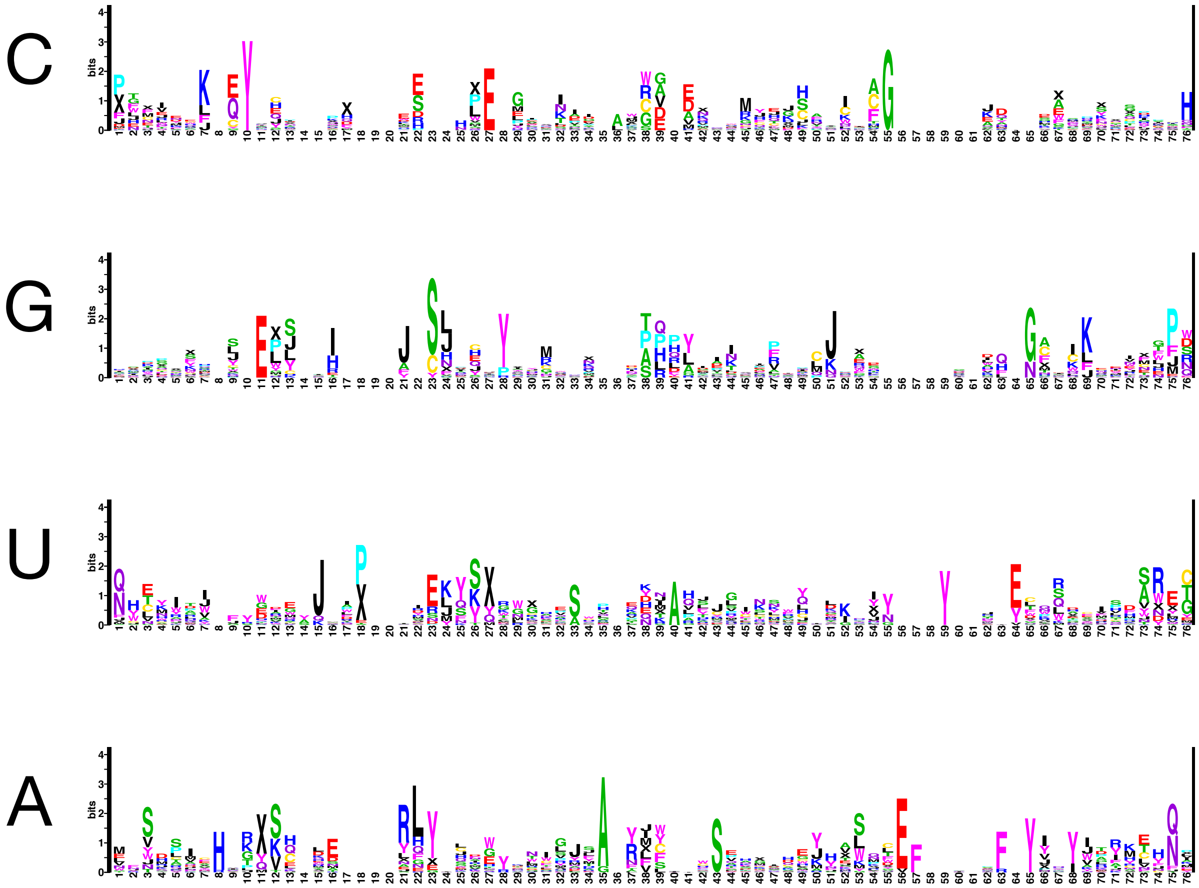

Figure S1: Function Logos for Cyanobacterial Clade A

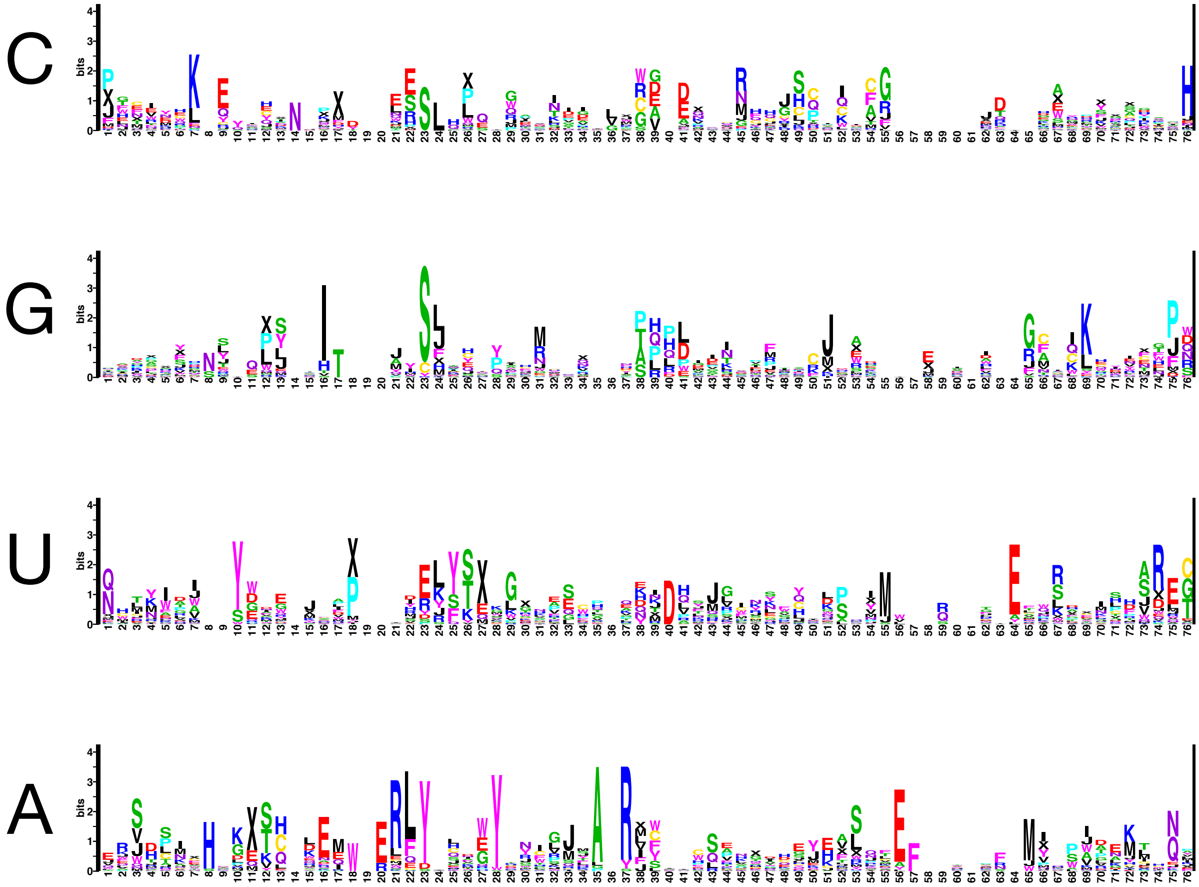

Figure S2: Function Logos for Cyanobacterial Clade B1

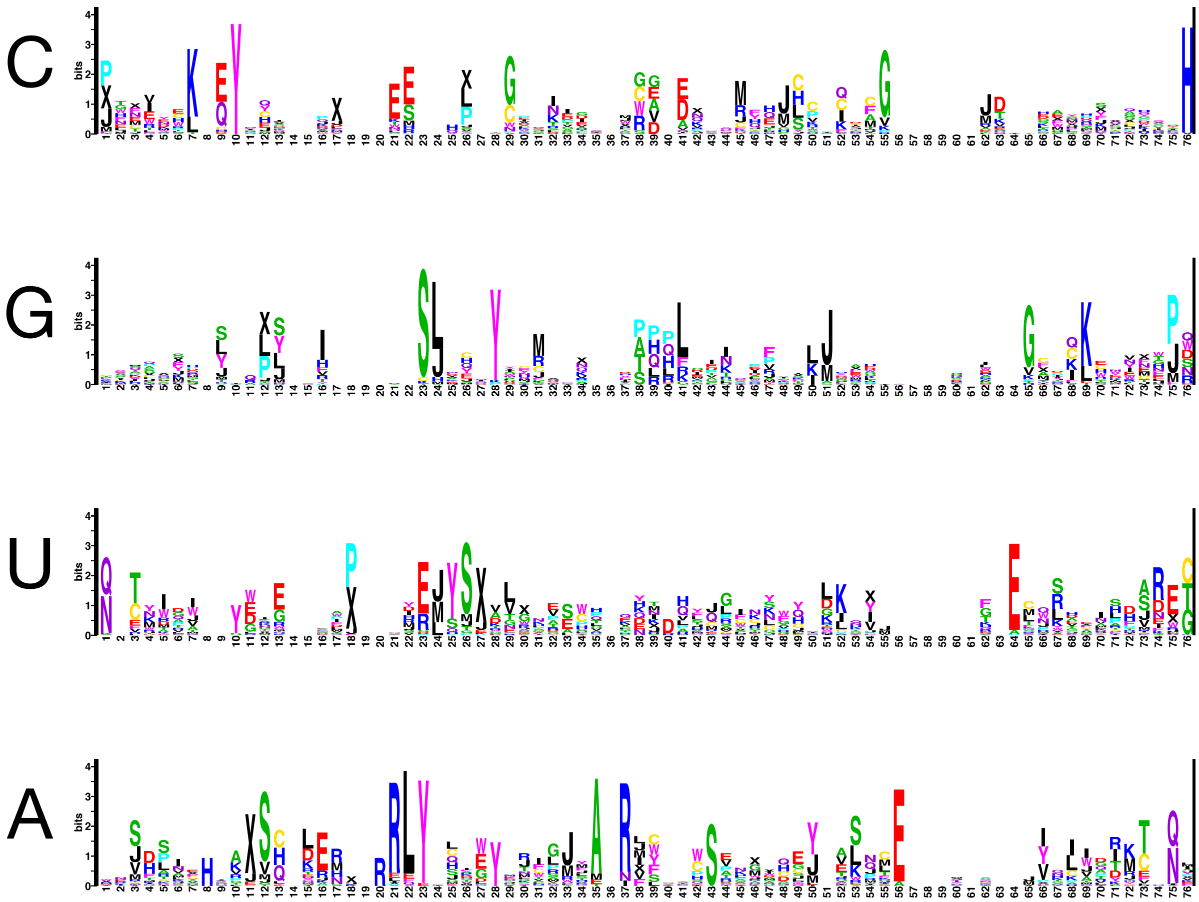

Figure S3: Function Logos for Cyanobacterial Clade B2+3

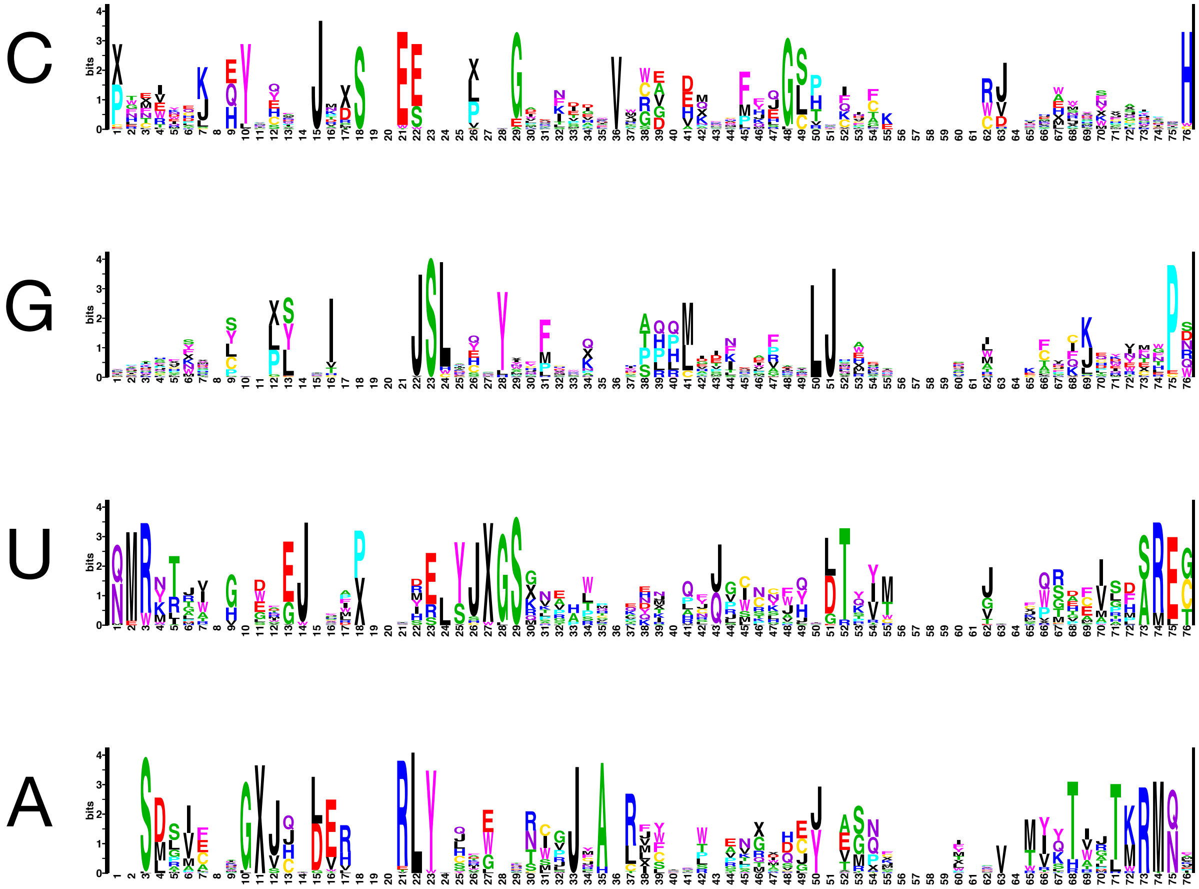

Figure S4: Function Logos for Cyanobacterial Clade C1

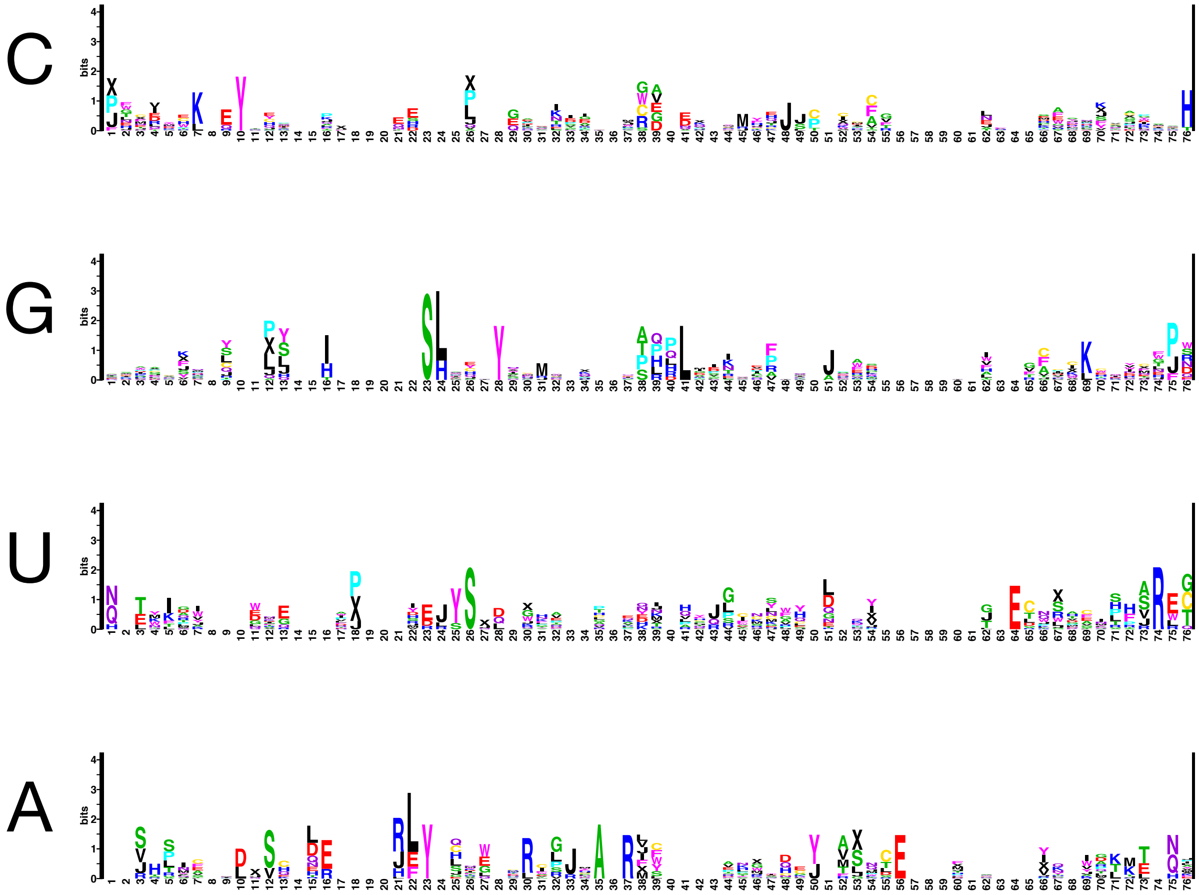

Figure S5: Function Logos for Cyanobacterial Clade C3

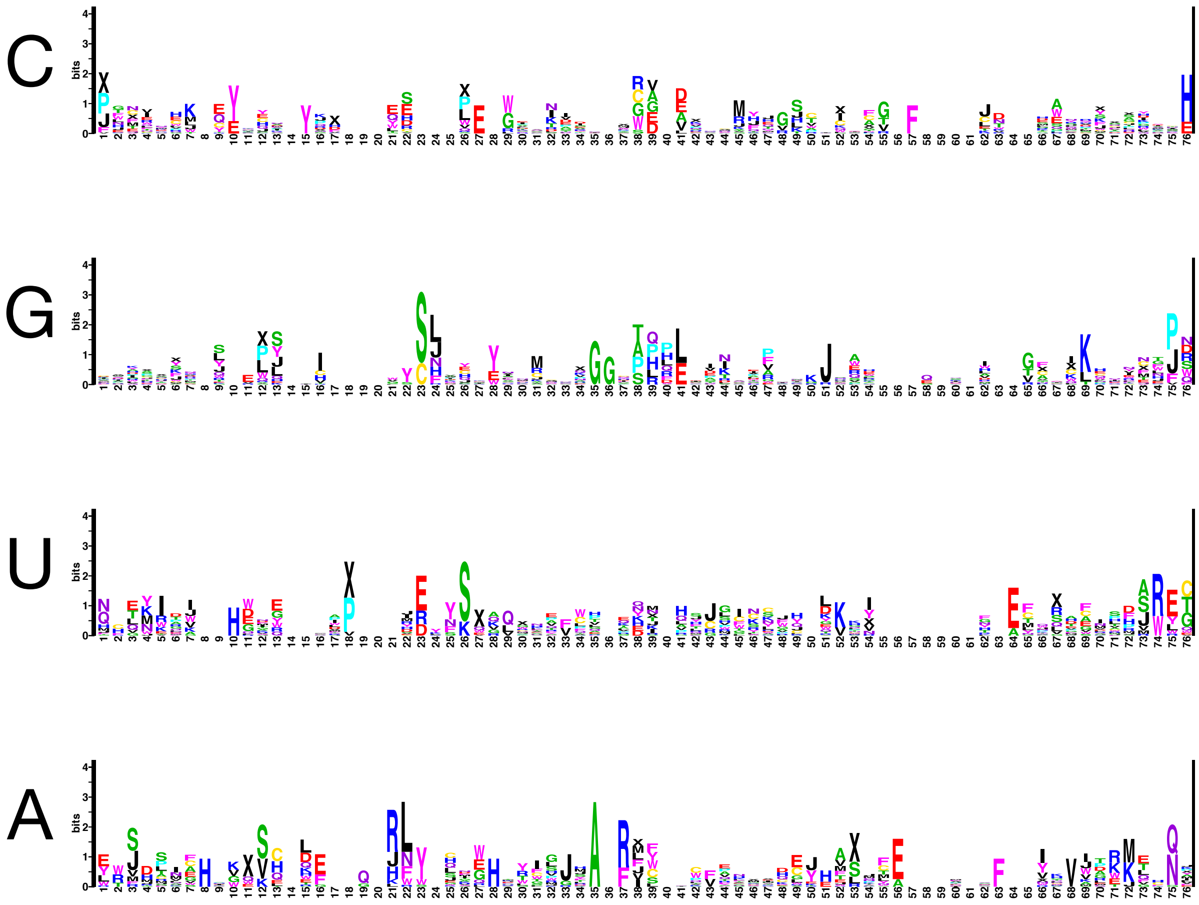

Figure S6: Function Logos for Cyanobacterial Clade E

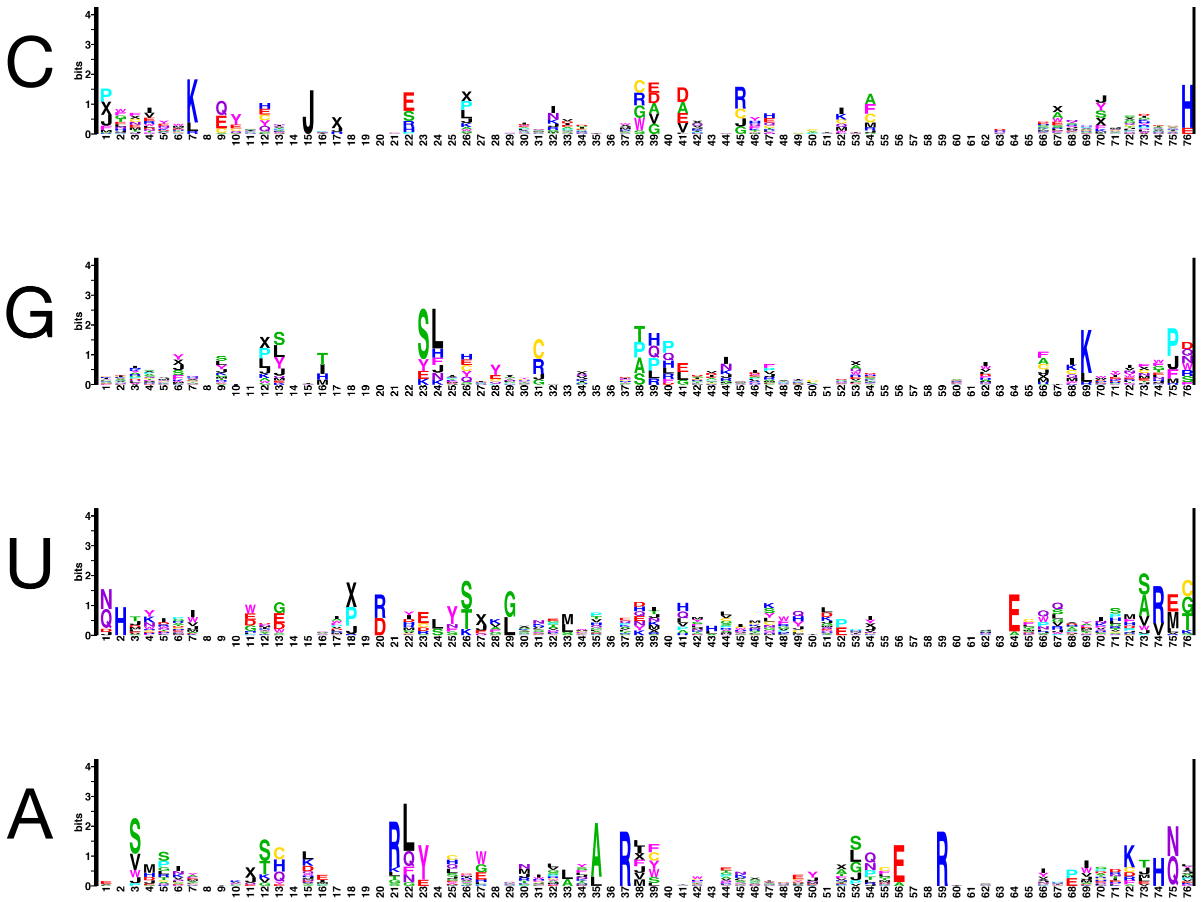

Figure S7: Function Logos for Cyanobacterial Clade F

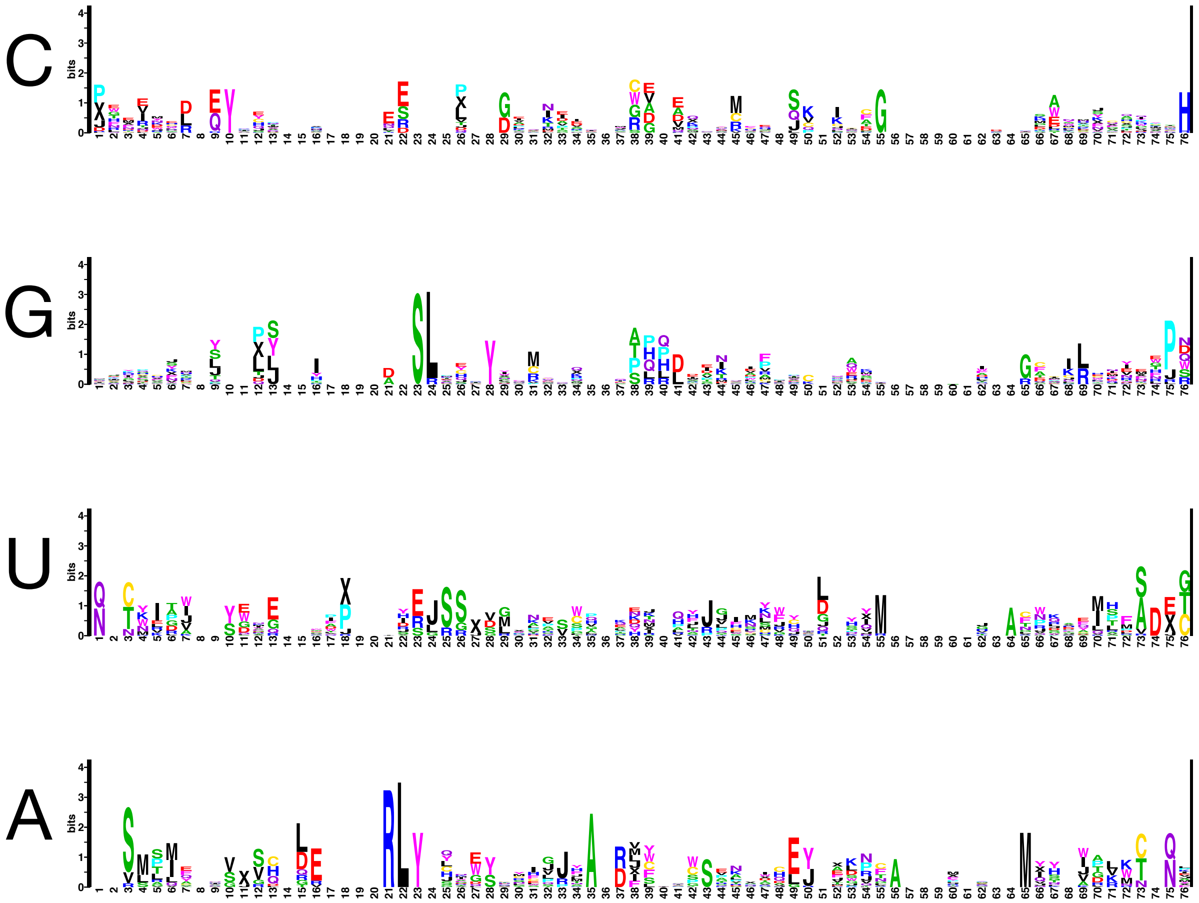

Figure S8: Function Logos for Cyanobacterial Clade G

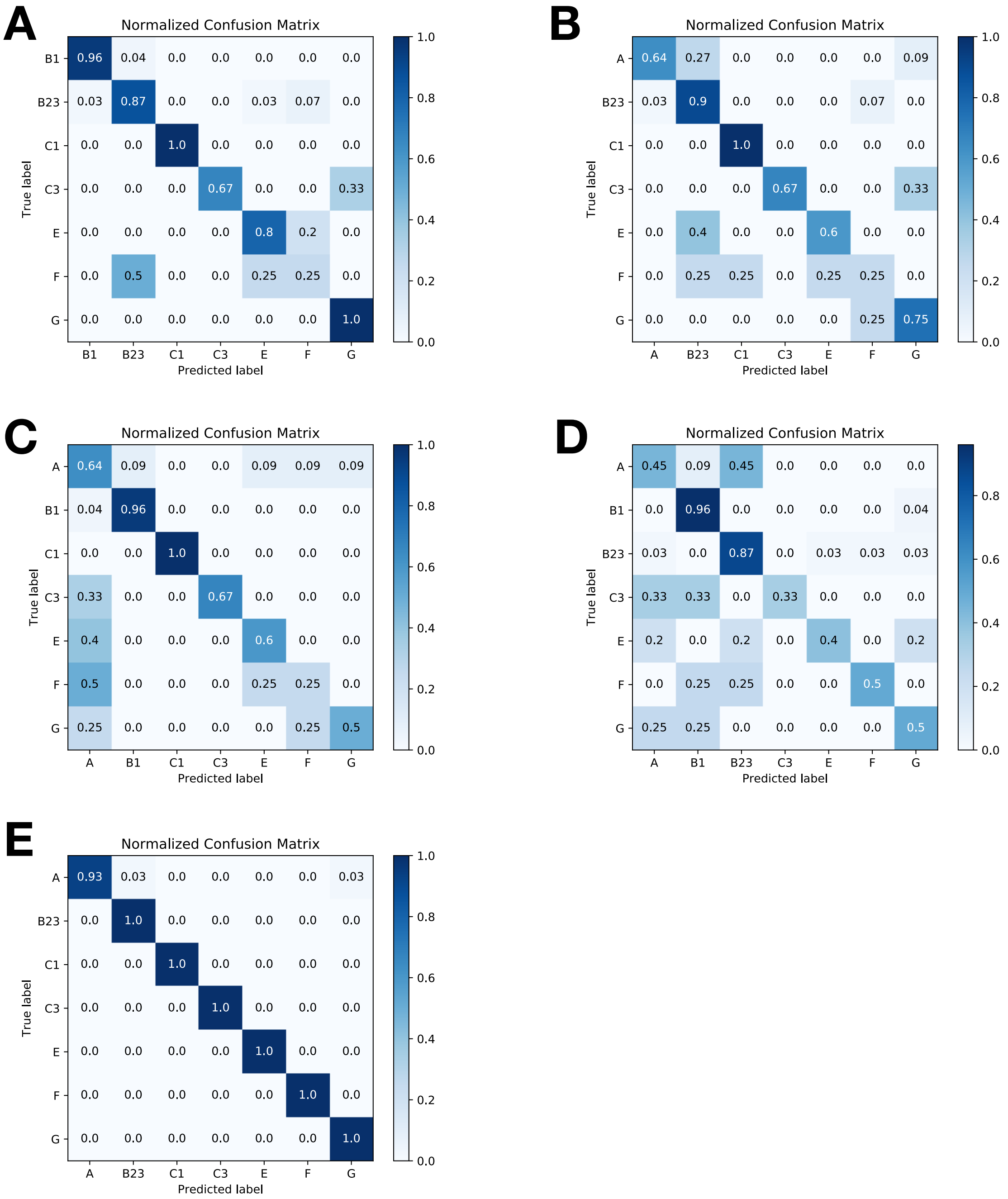

Figure S9: Normalized confusion matrices for (A) CYANO-MLP[!A], (B) CYANO-MLP[!B1], (C) CYANO-MLP[!B2+3], (D) CYANO-MLP[!C1], and (E) CYANO-MLP-BAL[!B1]. Where CYANO-MLP[!*X*] indicates variant of CYANO-MLP with clade *X* data excluded from training and BAL indicates clade balanced training data.

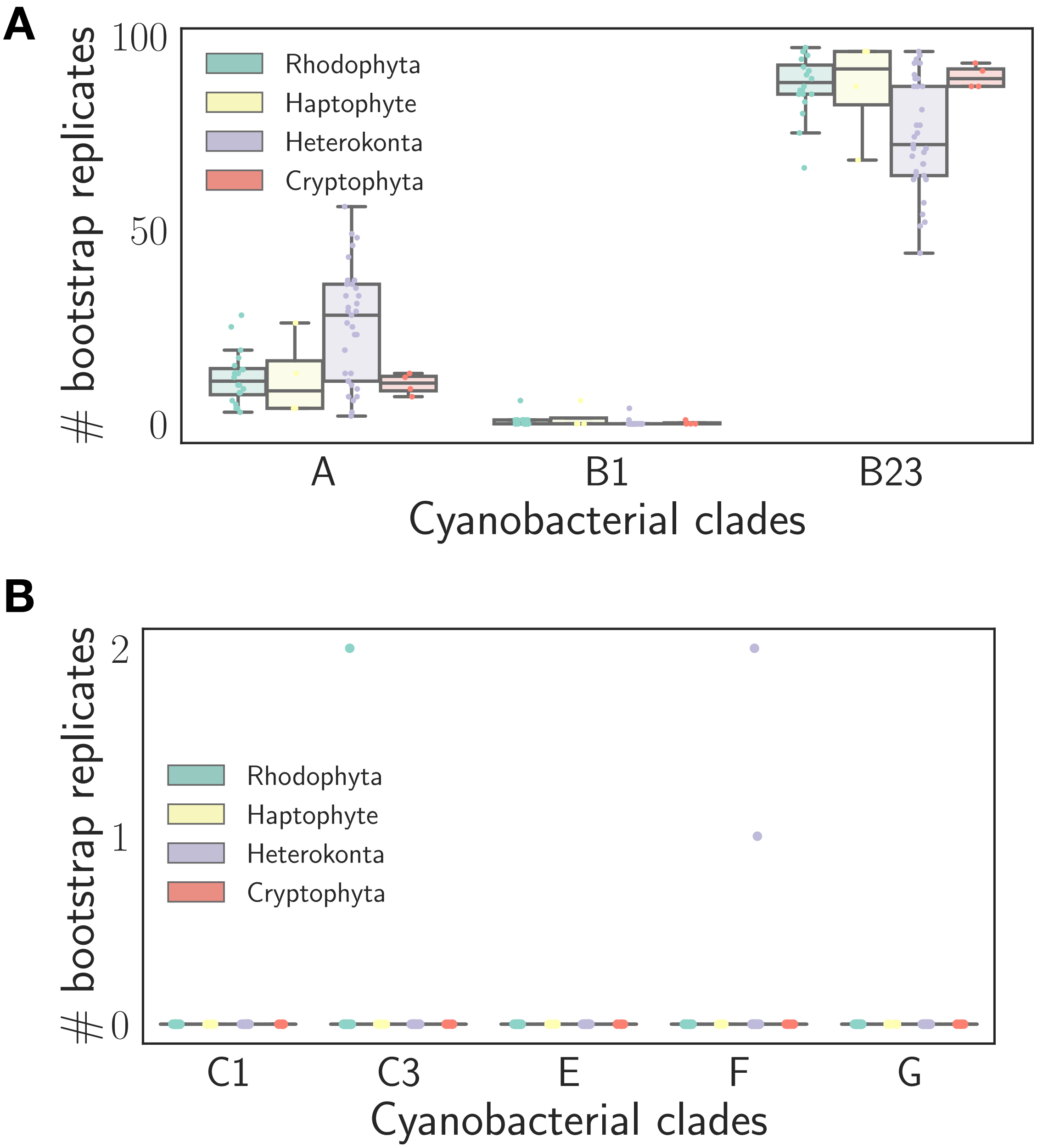

Figure S10: Classification results of 100 bootstrap replicates of each Rhodophyta derived plastid genome. Results are summarized by Red plastid group with boxes spanning from the 25th percentile (bottom) to the 75th percentile (top) of bootstrap replicates classifying to the indicated Cyanobacterial clade per genome with the bisecting line marking the median value. Error bars indicate the shorter of either ± the interquartile range or the span of bootstrap replicates per genome. Dots show bootstrap replicates for individual genomes. Plastid genome bootstrap replicates classifying to cyanobacteral clades (A) A, B1, B2+3, (B) C1, C3, E, F, or G.

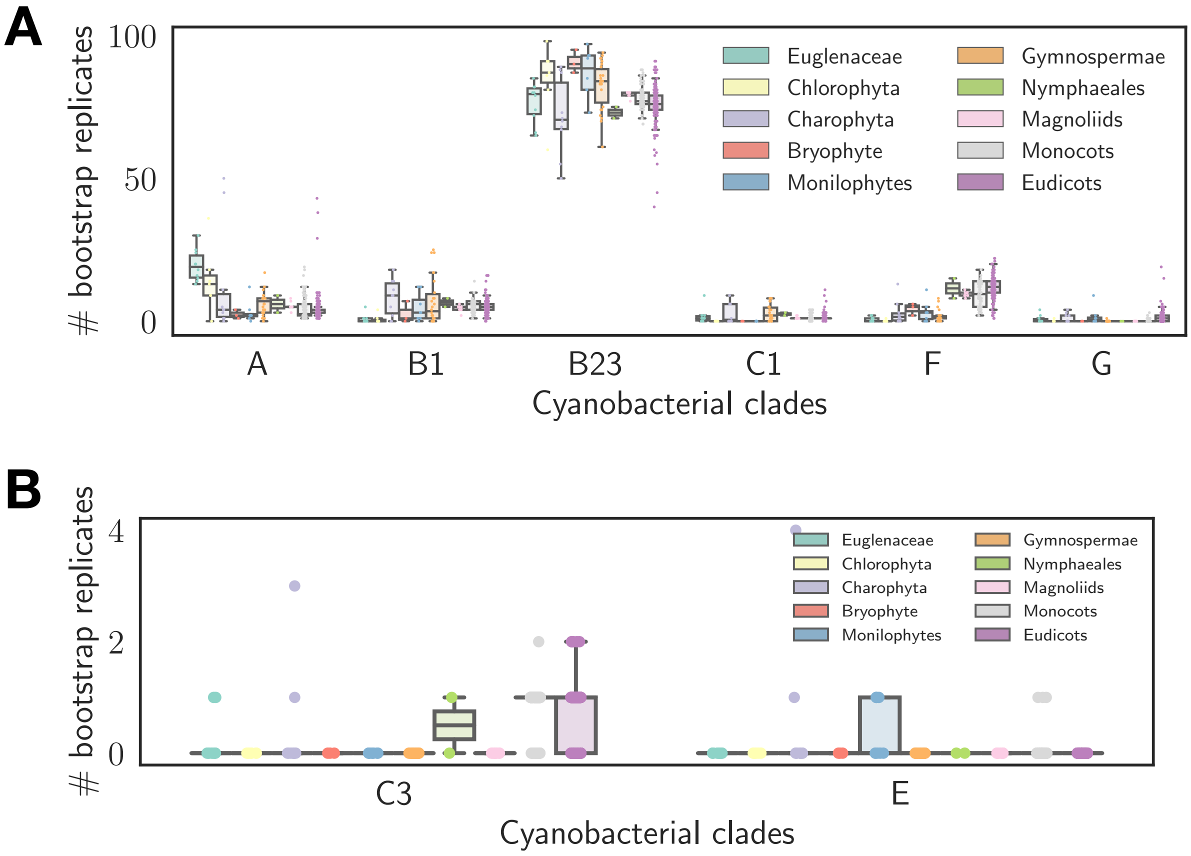

Figure S11: Classification results of 100 bootstrap replicates of each Chloroplastida derived plastid genome. (A) Bootstrap results for plastid genomes classifying to cyanobacterial clades A, B1, B2+3, C1, F, and G. (B) Bootstrap results for plastid genomes classifying to cyanobacterial clades C3 and E.

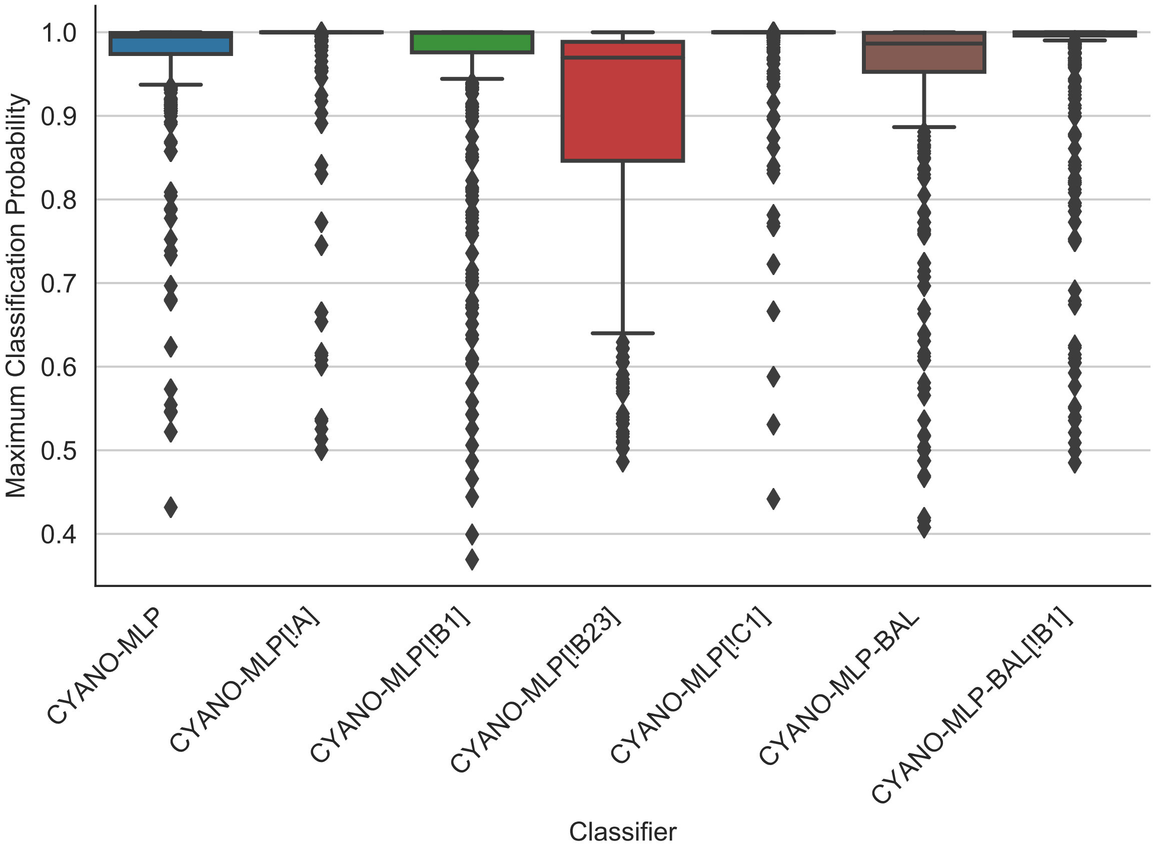

Figure S12: Box plot of maximum classification probability of each plastid genome. Error bars are the lesser of 1.5 IQR or full range of data. CYANO-MLP[!*X*] indicates variant of CYANO-MLP with clade *X* data excluded from training and BAL indicates clade balanced training data.
